# Sugar analog synthesis by *in vitro* biocatalytic cascade: A comparison of alternative enzyme complements for dihydroxyacetone phosphate production as a precursor to rare chiral sugar synthesis

**DOI:** 10.1371/journal.pone.0184183

**Published:** 2017-11-07

**Authors:** Carol J. Hartley, Nigel G. French, Judith A. Scoble, Charlotte C. Williams, Quentin I. Churches, Andrew R. Frazer, Matthew C. Taylor, Greg Coia, Gregory Simpson, Nicholas J. Turner, Colin Scott

**Affiliations:** 1 CSIRO Land and Water, Black Mountain Laboratories, Canberra, Australia; 2 CSIRO Manufacturing, Parkville, Melbourne, Australia; 3 School of Chemistry, CoEBio3, University of Manchester, Manchester, United Kingdom; Tianjin Institute of Industrial Biotechnology, CHINA

## Abstract

Carbon-carbon bond formation is one of the most challenging reactions in synthetic organic chemistry, and aldol reactions catalysed by dihydroxyacetone phosphate-dependent aldolases provide a powerful biocatalytic tool for combining C-C bond formation with the generation of two new stereo-centres, with access to all four possible stereoisomers of a compound. Dihydroxyacetone phosphate (DHAP) is unstable so the provision of DHAP for DHAP-dependent aldolases in biocatalytic processes remains complicated. Our research has investigated the efficiency of several different enzymatic cascades for the conversion of glycerol to DHAP, including characterising new candidate enzymes for some of the reaction steps. The most efficient cascade for DHAP production, comprising a one-pot four-enzyme reaction with glycerol kinase, acetate kinase, glycerophosphate oxidase and catalase, was coupled with a DHAP-dependent fructose-1,6-biphosphate aldolase enzyme to demonstrate the production of several rare chiral sugars. The limitation of batch biocatalysis for these reactions and the potential for improvement using kinetic modelling and flow biocatalysis systems is discussed.

## Introduction

Carbon-carbon ligations are among the most important reactions in modern organic synthetic chemistry. Although there are a range of reactions that can be used to achieve C-C coupling, biocatalysis can be used to provide exquisite chiral purity at newly formed stereocentres [1–3]. In biocatalysis, it is the aldolase enzymes (E.C. 4.1.2.x) that have been most extensively exploited to provide this catalytic function [1].

**Fig 1:**
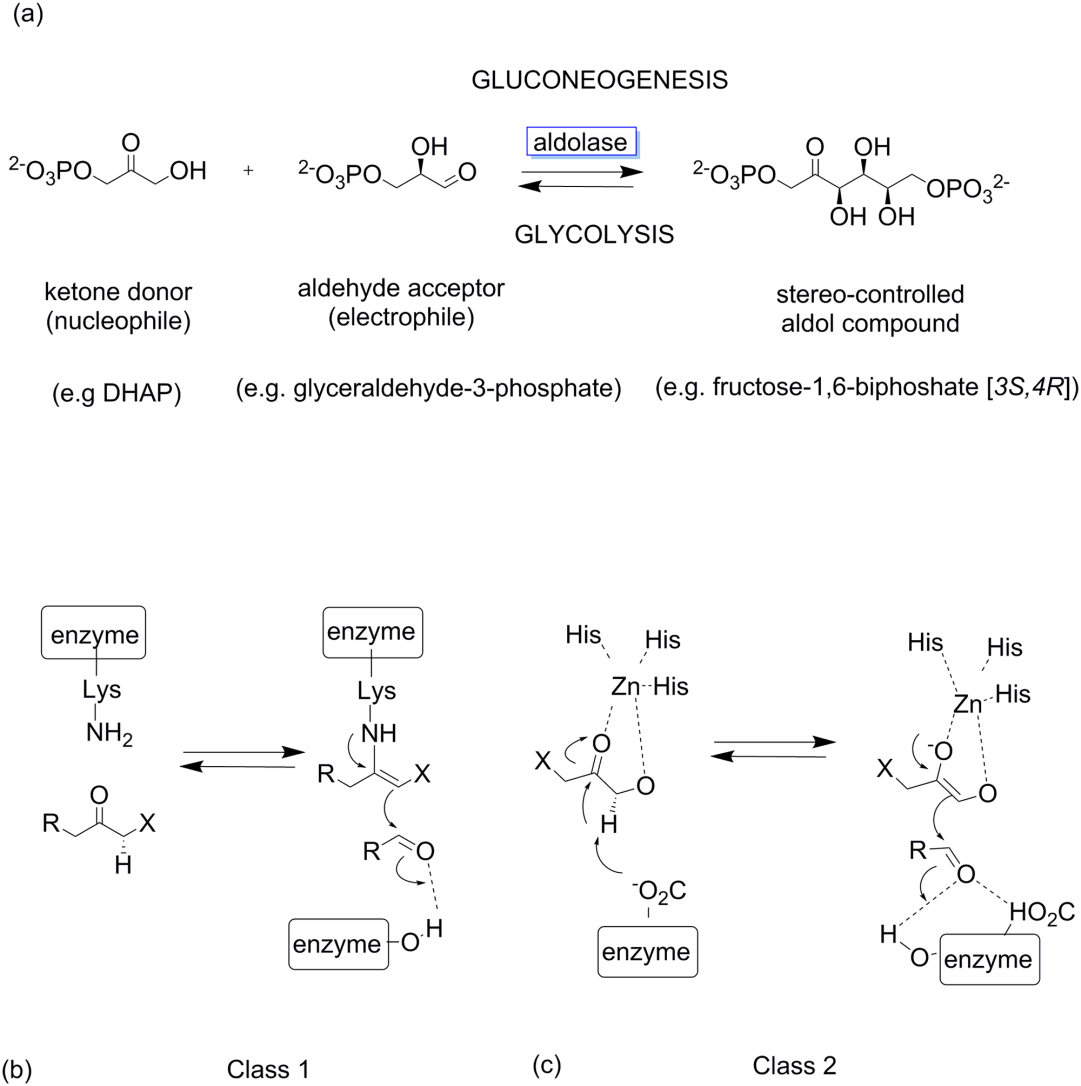
**Aldolase enzymes are involved in both gluconeogenesis and glycolysis (a) and are classified into two classes by two distinct mechanisms of action for nucleophilic activation**. Class I aldolases exhibit a strictly conserved lysine residue which forms a covalent Schiff base with the donor molecule to generate an enamine nucleophile (b) whilst class II aldolases use a divalent metal cation cofactor to promote enolization of the donor molecule *via* bidentate Lewis acid complexation (c).

In biology, aldolases promote both aldol coupling reactions in anabolic processes, such as gluconeogenesis, and retroaldol reactions in catabolic processes such as glycolysis (Fig 1a). The aldolases are categorized by their substrate requirements and their reaction mechanisms. Class I aldolases use a conserved catalytic lysine to form a covalent Schiff base intermediate with the donor aldehyde and generate an enamine nucleophile, which is then displaced by the acceptor aldehyde, thus regenerating the lysine residue, while class II aldolases have a catalytic zinc ion that forms a zinc enolate intermediate with the donor aldehyde that attacks the acceptor aldehyde in a C-C bond forming reaction (Fig 1b). The identity of the donor aldehyde is also used to classify aldolases, with aldolases dependent upon dihydroxyacetone phosphate (DHAP), pyruvate, phosphoenolpyruvate, acetaldehyde or glycine [1].

The DHAP-dependent aldolases are of particular interest; they produce two new stereocentres as a result of C-C coupling, and there are stereo-complementary DHAP-dependent aldolases available that can each produce one of the four possible stereoisomers with excellent enantio- and diastereo-selectivity [4]. This class of aldolases has also been shown to have a relatively relaxed acceptor aldehyde specificity, providing access to a wide range of synthons[5]. However, the donor aldehyde (DHAP) is unstable and it would be preferable to generate it *in situ* for maximal process efficiency; chemical synthesis routes are also complex, inefficient and expensive, involving many protection and deprotection steps and the use of toxic reagents [6, 7].

Several enzymatic routes have been investigated for the *in situ* production of DHAP [6], and generally follow three main options:

1. the retroaldol reaction catalyzed by DHAP-dependent aldolase (e.g. fructose-1,6-biphosphate aldolase enzyme FruA) to liberate DHAP from fructose-1,6-phosphate[8], using triose phosphate isomerase (TPI) to convert the alternative product glyceraldehyde-3-phopshate to DHAP
2. the conversion of glycerol to dihydroxyacetone (DHA) by glycerol dehydrogenase (EC 1.1.1.6) and then conversion of DHA to DHAP by dihydroxyacetone kinase (EC 2.7.1.29)
3. the conversion from glycerol to glycerol-3-phosphate by glycerol kinase (EC 2.7.1.30) and then conversion of glycerol-3-phosphate to DHAP by either glycerol-3-phosphate dehydrogenase (EC 1.1.1.8) or glycerophoshate oxidase (EC 1.1.3.21).

**Fig 2:**
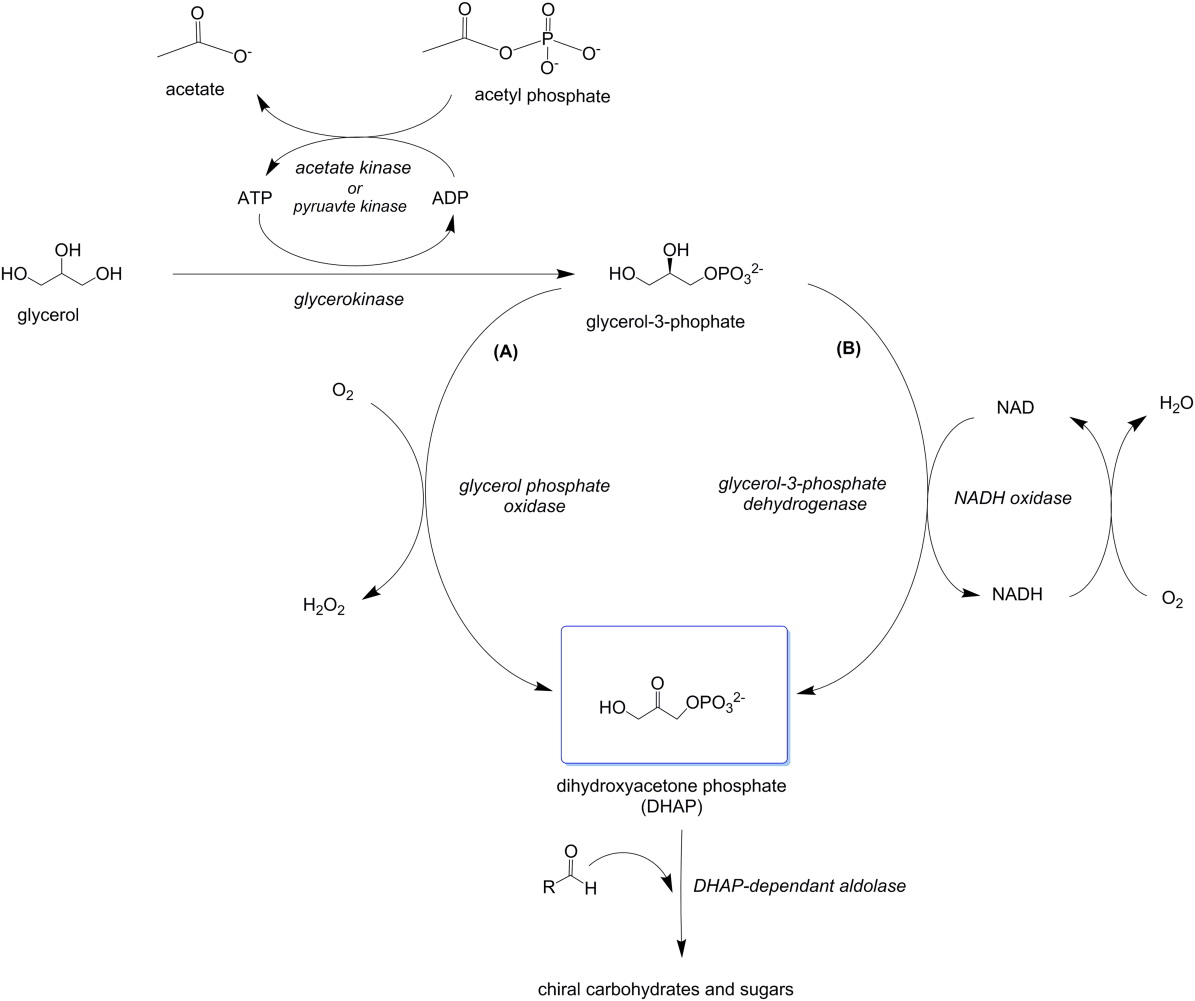
Enzymatic cascades for the conversion of glycerol to DHAP via glycerol-3-phosphate. Glycerol is converted to glycerol-3-phosphate by a glycerol kinase enzyme with concomitant regeneration of ATP by an acetate or pyruvate kinase enzyme. The glycerol-3-phopshate is then oxidized to DHAP by either an *L*- glycerol-3-phosphate oxidase enzyme (A) or a glycerol-3-phosphate dehydrogenase enzyme (B).

The retroaldol reaction coupling FruA with triose phosphate isomerase (TPI) is advantageous as the fructose-1,6-biphosphate starting substrate can be cheaply generated from sucrose, glucose or fructose using a five-enzyme cascade, but this synthesis option is complicated by the requirement for laborious purification of a large number of enzyme catalysts [5, 9], albeit choosing or engineering high-activity enzymes can alleviate this complication [10–12]. Whilst the simplest of these is the conversion of DHA to DHAP, the yields of DHAP from DHA reported to date remain low for preparative synthesis, or require compatible ATP regeneration systems [6, 13, 14]. Although requiring two enzymatic steps, as opposed to the single enzymatic step needed for the conversion of dihydroxyacetone (DHA) to dihydroxyacetone phosphate (DHAP) by glycerol or dihydroxyacetone kinase enzymes, the conversion of glycerol to DHAP via glycerol-3-phosphate has the advantage of producing a stable, storable intermediate compound in glycerol-3-phosphate. Glycerol-3-phosphate can then be rapidly oxidized to DHAP by either an NAD-dependent glycerol-3-phosphate dehydrogenase or glycerol phosphate oxidase enzyme, for *in situ* generation of DHAP as a substrate for DHAP-dependent aldolase catalytic reactions ([5], Fig 2). Cascades that initiate with the phosphorylation of glycerol *via* phytase or alkaline/acid phosphatases and are followed by the oxidation of the resultant glycerol phosphate to DHAP by glycerol phosphate oxidase have also been studied[15–17]. However, phosphorylation of glycerol by both alkaline or acid phosphatase and phytase results in a racemic mixture of glycerol-3-phosphate (albeit a nine fold excess of *L*-glycerol-3-phosphate is achievable with calf intestinal alkaline phosphatase), resulting in less efficient conversion of glycerol to DHAP [7, 18–20].

Herein, we compare a series of enzymes, sourced from a range of organisms, for the construction of an *in vitro* enzymatic cascade for the conversion of glycerol to DHAP *via* glycerol-3-phosphate. We have first examined the phosphorylation of glycerol by glycerol kinase, with concomitant ATP regeneration by acetate kinase enzymes (Fig 2). Secondly, we have examined two divergent enzymatic routes for the conversion of glycerol-3-phosphate to DHAP, using either a flavoprotein glycerophosphate oxidase enzymes (with catalase to mitigate hydrogen peroxide by-product) or NAD(P)-dependant glycerol-3-phosphate dehydrogenases with concomitant regeneration of NAD by water-forming NADH oxidase enzymes (Fig 2). The most efficient enzyme cascades for DHAP production were then coupled with a stereoselective DHAP-dependent aldolase enzyme (FruA) in a multi-enzyme cascade to produce a variety of chiral carbohydrates with potential applications as pharmaceutical synthons.

## Methods and materials

### DNA manipulation

The pETScFruA vector which encodes the DHAP-dependent fructose-1,6-biphosphate aldolase enzyme from *Staphylococcus carnosus* was a gift from A. Frazer (University of Manchester, Manchester, UK). For all other constructs the gene of interest was synthesized by GeneArt (Thermofisher, Germany), and cloned into either pETCC2 ([21]; modified pET14b, Novagen) using *Nde*I and IHI/*Eco* RI sites, or into pDEST17 using Gateway and Clonase II techniques (Thermofisher Life Sciences) (see Supporting Information for further details). All constructs were confirmed by DNA sequencing (Macrogen, S. Korea).

### Protein expression and purification

With two exceptions, enzymes were obtained by cloning, expression and purification from *E*. *coli* cells. Briefly, synthetic genes were transferred into either pDEST17 or pETCC2, transformed into *E*.*coli* BL21AI or *E*.*coli* BL21DE3* (Invitrogen) cells, respectively. Cells were cultured overnight at 37°C then induced for 2, 4, 6 or 24 hours with either arabinose or IPTG (0.2 M and 1 mM final concentration, respectively) and then harvested, resuspended in one tenth volume resuspension buffer (50mM Tris-Cl, 250mM NaCl, pH7.5) and lysed with Bugbuster (Novagen). Protein expression was analyzed by SDS-PAGE separation and stained with NuBlue (Novagen). The optimal expression time was selected and large scale expression cultures of 1–2 L prepared in the same way as above, followed by purification of HIS-tagged protein by elution with resuspension buffer (50mM Tris-Cl, 250mM NaCl, pH7.5) containing increasing concentration of imidazole from Ni-sepharose (HIS-TRAP, GE Healthcare). The desired protein fractions were then pooled and further purified using a Superdex 200 size exclusion column (GE Healthcare). Pooled fractions were then concentrated and stored at 4°C, or -80°C, as required.

### Enzymatic assays

Glycerol kinase assays were performed at room temperature in 1 mL volume essentially as described by Pettigrew, 2009 [22], but with direct detection of ADP (Sigma-Aldrich, USA) and ATP (Sigma-Aldrich, USA) by HPLC analysis of the reaction (see Analytical Methods below). A typical reaction contained 1 mM glycerol, 10 mM MgCl_2_, 50 mM NaHCO_3_ buffer (pH 9.2), 1 mM ATP with approximately 2μg/mL enzyme (35 nM). Kinetics were determined by varying the concentrations of ATP or glycerol whilst maintaining the other in excess, and kinetic determinants calculated using Hyper^™^ (J.S. Easterby, Liverpool University). Substrate and cofactor concentrations ranged from 0.1 to 10 x *K*_M_.

Acetate kinase assays were conducted in the same manner, replacing ATP with ADP and glycerol with acetyl phosphate (10mM unless otherwise stated) or phosphoenol pyruvate (10mM unless otherwise stated). Kinetics were determined by varying the concentrations of ADP or acetyl phosphate or phosphoenol pyruvate whilst maintaining the other components in excess, and kinetic determinants calculated using Hyper^™^ (J.S. Easterby, Liverpool University). Substrate and cofactor concentrations ranged from 0.1 to 10 x *K*_M_.

Glycerol-3-phosphate dehydrogenase were conducted essentially as described by Sakasegawa *et al*., 2004 [23], by calculating the increase in NADH from absorbance at 340 nm. A typical reaction comprised 1 mM glycerol-3-phosphate, 50 mM NaHCO_3_ buffer (pH 9.2), 1 mM NAD^+^ with approximately 2μg/mL enzyme (54 nM). Oxidation of NADH by NADH oxidase enzymes was calculated by measuring the decrease in absorbance at 340 nm. A typical NADH oxidase reaction comprised 1 mM NADH, 50 mM NaPO_4_ buffer (pH 7.0), with approximately 2 μg/mL enzyme (42 nM). Kinetics were determined by varying the concentrations of NAD(P)/NAD(P)H or glycerol-3-phosphate, whilst maintaining the other components in excess, and kinetic determinants were calculated using Hyper^™^ (J.S. Easterby, Liverpool University). Substrate and cofactor concentrations ranged from 0.1 to 10 x *K*_M_.

Glycerophosphate oxidase activity was measured by coupling the peroxide generated from oxidation of glycerol-3-phosphate with horseradish peroxidase (HRP, P8375, Sigma-Aldrich, USA) and monitoring HRP activity using colorimetric reaction with 2,2′-Azino-bis(3-ethylbenzothiazoline-6-sulfonic acid) diammonium salt (ABTS; Roche, Switzerland). A typical 200μL reaction comprised 100μL of 0.4mg/mL ABTS in 0.2M phosphate buffer pH7.0, 7.5U/mL HRP, glycerol-3-phosphate substrate (varying concentrations), 286 pmoles of GlpO enzyme. The absorbance at A_420nm_ was recorded for at least ten minutes at room temperature, and the equivalent activity calculated from the extinction coefficient of ABTS (36.8 mM^-1^cm^-1^).

Paired reactions were conducted in 50 mM NaPO_4_-citrate buffer pH 7.9. with 10 mM glycerol, 10mM acetyl phosphate co-substrate, 100 μM ATP (Pair 1) or 50 mM NaPO_4_-citrate buffer pH 7.9. with 10 mM glycerol-3-phosphate, 100 μM NAD^+^ (Pair 2). Enzyme loadings were normalised to *k*_cat_ values obtained (see Table 1), estimating loading to ensure that cofactor-recycling was at least one order of magnitude faster than the catalytic reaction resulting in the desired product, in order to drive the rate towards optimal product yield. Thus individual enzyme loadings in pmoles per paired reaction were as follows. Pair 1: GlpK_*Tk*_− 14.32; GlpK_*Bs*_− 89; AceK_*Ms*_− 11.25; AceK_*Ms*_− 20.1. Pair 2: G3PD_*Ec*_ -82.2; G3PD_*Af*_—155; G3PD_*Oc*_ -76; G3PD_*Ms*_ -82.2; NOX_*Ca*_− 10.1; NOX_*Ls*_− 27 pmole per reaction.

**Table 1:** Steady state kinetics for enzymes involved in conversion of glycerol to DHAP.

Enzyme	Substrate	*K*_M_ (μM)	*k*_cat_ (s^-1^)	*k*_cat_/ *K*_M_ (M^-1^s^-1^)	pH Optimum	pH Range	Ref.
GlpK_*Cb*_	glycerol	356 ± 27	93	2.6 x 10^5^	9.5	7.0–9.5	This study
	ATP	135 ± 16		6.9 x 10^5^			
GlpK_*Tk*_	glycerol	15.4 ± 2	940	6.1 x 10^7^	8.5	7.0–9.5	[25]
	ATP	111 ± 12		8.4 x 10^6^			
GlpK_*Bs*_	glycerol	153 ± 17	150	9.8 x 10^5^	9.5	7.0–9.5	Sigma-AldrichG0774
	ATP	125 ± 11		1.2 x 10^6^			
AceK_*Ms*_	ADP	113 ± 9	1103	9.8 x 10^6^	7.5	7.0–9.0	This study
	AcP	390 ± 8		2.8 x 10^6^			
AceK_*Mt*_	ADP	80 ± 5	2690	3.4 x 10^7^	7.4	7.0–9.0	[31]
	AcP	470 ± 34		5.7 x 10^6^			
PyrK_*Bs*_	ADP	550 ± 51	587	1.1 x 10^6^	7.5	7.0–9.0	Sigma P1903
	PEP	100 ± 11		5.9 x 10^6^			
G3PD_*Ms*_	NADP^+^	258 ± 21	85	3.3 x 10^5^	9.0	7.0–9.5	This study
	G3P	59 ± 5		1.4 x 10^6^			
G3PD_Ec_	NAD^+^	158 ± 24	85	5.4 x 10^5^	9.0	7.0–9.5	[27]
	G3P	59 ± 4		1.4 x 10^6^			
G3PD_*Af*_	NADP^+^	800 ± 76	196	2.5 x 10^5^	9.5	6.6–9.5	[23]
	G3P	111 ± 12		1.8 x 10^6^			
G3PD_*Oc*_	NAD^+^	804 ± 56	595	7.4 x 10^5^	9.0	7.5–9.0	Sigma G6880
	G3P	136 ± 9		4.4 x 10^6^			
NOX_*Ca*_	NADH	258 ± 21	1252	4.9 x 10^6^	7.0	5.0–9.0	[28]
	NADPH	ND					
NOX_*Ls*_	NADH	6.7 ± 0.9	1965	2.9 x 10^8^	7.5	5.0–9.0	[29]
	NADPH	6.1 ± 0.6	550	9.0 x 10^7^			
GlpO_*Ec*_	G3P	968 ± 25	81.2	8.4 x 10^4^	7.0	5.0–6.7	[26]
GlpO_*Mg*_	G3P	140 ± 24	1395	9.9 x 10^6^	7.0	5.0–7.0	This study

Coupled cascade reactions for the conversion of glycerol to DHAP via glycerol-3-phosphate were performed at room temperature (22°C) in a total volume of 1ml comprising 50 mM NaPO_4_-citrate buffer pH 7.9 with 10 mM glycerol, 10mM acetyl phosphate co-substrate, 100 μM ATP and 100 μM NAD^+^. Individual enzyme loading amounts were as used for paired reactions above, except that catalase (Sigma-Aldrich 60634; final concentration 3U per μL) was added to reactions utilising GlpO, in order to mitigate the production of excess hydrogen peroxide.

Production of chiral sugars using a five step enzymatic cascade was performed at room temperature (22°C) in a total volume of 1ml comprising 50 mM NaPO_4_-citrate buffer pH 7.9 with 10 mM glycerol, 10mM acetyl phosphate co-substrate, 100 μM ATP and 100 μM NAD^+^. Individual enzyme loading amounts were as used for the paired reactions above. After reaction for one hour, aldolase enzyme FruA_*Sc*_ (Supporting Information; final concentration 1.5 nmoles) was added together with 10mM acetaldehyde or 10mM glyceraldehyde-3-phosphate. The reaction was further incubated at room temperature (22°C), and the accumulation of aldol product measured using LC-MS as described in analytical methods below.

### Analytical methods

#### HPLC separation of ATP and ADP

HPLC separation was conducted using an Agilent Eclipse XDB column (50 mm x 4.6mm) with an isocratic gradient of 75% solvent A and 25% solvent B. Solvent A: 20 mM tetrabutylammonium phosphate (TBAP) in 10 mM ammonium phosphate buffer pH 4.0; solvent B: acetonitrile. Flow rate 1 mL/min, detection at 240 nm using diode array detector (Agilent Technologies, USA). Peaks eluted as follows: ADP 1.2 mins, ATP 1.8 mins.

#### LCMS analysis of glycerol-3-phosphate (G3P), dihydroxyacetone phosphate (DHAP) and aldol products

G3P and DHAP were separated using a modification of the method described in Prieto-Blanc *et al*., 2010 [24]. Chromatographic conditions were SIELC ObeliscN column (100 mm x 2.1 mm) with 20% mobile phase A, 80% mobile phase B for 5 minutes. Mobile phase A: 25 mM ammonium formate pH 4.0; mobile phase B: acetonitrile. Mass spectrophotometric detection was conducted using API-ES negative mode with an Agilent 6120 Quadropole LCMS. Glycerol-3-phosphate was quantified by selected ion monitoring of ion [M]^-^ *m/z* = 171, DHAP quantified by selected ion monitoring of ion [M]^-^ *m/z* = 169, after establishing suitable selected ions using positive and negative scanning of standards. Quantitation was based on comparison to standard calibration curves produced in the same manner. Fructose-1,6-biphosphate was quantified by comparison with known standard calibration curves using selected ion monitoring of ion [M]^+^ *m/z* = 341.116.

#### GCMS analysis of glycerol, glycerol-3-phosphate (G3P) and dihydroxyacetone phosphate (DHAP)

All three compounds could be separated and detected after derivatization with *N*-Methyl-*N*-(trimethylsilyl)trifluoroacetamide (MSTFA) in pyridine (Sigma-Aldrich, USA). Samples were snap frozen in liquid nitrogen and then freeze-dried overnight. The resultant powder was resuspended in 50 μL 240 mM methoxyamine-HCl (Sigma-Aldrich, USA) in pyridine. After incubation at 65°C for 50 minutes, 80 μL of MSTFA was added and the samples were incubated at 65°C for a further 50 minutes. Samples were centrifuged at 10 000 x *g* for 10 mins, and GC-MS separation was performed with a HP5-MS column (Agilent Technologies, USA) using the following program: Carrier gas helium at 30mL per minute. Oven program: 1 mL/min at 100°C, hold for 0.2 min then ramp at 10°C/min to 250°C and hold for 10 min. Injector temperature: 280°C. Injection volume: 1 μL. Products were detected by selected ion monitoring for DHAP (*m/z* ions 400, 315, 299, 73), G3P (*m/z* ions 357, 299, 73) and glycerol (*m/z* ions 205, 147, 73).

## Results and discussion

### Selection of suitable enzymes

A total of twenty-four candidate enzymes were selected for the four enzymatic reactions required to convert glycerol to glycerol-3-phosphate and then DHAP (S1 Table) Initial selection of candidate enzymes was based on reported catalytic efficiency, combined with suitable pH range and temperature optima for use in multi-enzyme cascade reactions i.e. activity within the range of pH 7.0–9.0 and between 25–37°C. Most of the selected enzymes had known crystal structures (those with PDB accession numbers in S1 Table), but some potentially suitable but some previously uncharacterised enzyme candidates were also included to expand the range of enzymes for assessment. Ten of the candidate enzymes were eliminated due to poor levels of expression in the heterologous expression organism (*E*. *coli* BL21 AI or DE3*; see S1 Table and S1 File for details), the remaining fourteen were then selected for further characterization in multi-enzyme cascades.

Eight initial glycerol kinase enzymes candidates were selected based on detailed characterisation reported in the literature (GlpK_*Ec*_ and GlpK_*Sc*_), thermostability and catalytic efficiency (GlpK_*Tk*_, GlpK_*Hv*_ and GlpK_*Bs*_), as well as three previously uncharacterized glycerol kinases from *Clostridia* and *Mycobacteria* (GlpK_*Cb*,_ GlpK_*Ms6229*_ & GlpK_*Ms6756*_) (S1 Table). Three ATP-dependent glycerol kinases (GlpK) obtained from *Clostridium beijerinckii* (GlpK_*Cb*_; Acc. No. AIU00287; previously uncharacterised), *Thermococcus kodakarensis* (GlpK_*Tk*_; Acc. No. O93623; PDB 2ZF; [25]) and *Bacillus stearothermophilus* (GlpK_*Bs*_; Sigma G0774) were successfully expressed in *E*. *coli* BL21 (S1 Table) or obtained from Sigma -Aldrich. All three were soluble and active. Three ATP-recycling enzymes, two acetate kinases (AceK) from *Mycobacterium smegmatis* and *Methanosarcina thermophila* (AceK_*Ms*_; WI_011727188; PDB 4IJN and AceK_*Mt*_; WP_048129005; PDB 1TUU) and a pyruvate kinase (PyrK_*Bs*;_ Acc.No. WP_033014443; PDB 2E28) from *Bacillus stearothermophilus*, were also successfully expressed in *E*. *coli* BL21 (S1 Table).

The oxidation of glycerol-3-phosphate to DHAP was investigated using two flavin-dependent glycerophosphate oxidases (GlpO) from heme—deficient Lactobacilli strains, namely *Enterococcus casseliflavus* (GlpO_*Ec*_; Acc. No. WP 005237472; [26]) and *Mycoplasma gallisepticum* (GlpO_*Mg*_; Acc. No. WP 014574362; previously uncharacterized). GlpO uses molecular oxygen to re-oxidize the flavin cofactor after catalysis, which produces hydrogen peroxide as a by-product. The presence of the non-specific oxidant in the reaction mixture could lead to oxidative inactivation of the enzymes, thereby limiting the effective duration of the reaction, and could also lead to the generation of additional unwanted oxidation products that may need to be removed by an additional purification step. This problem can be mitigated by the addition of catalase, albeit at additional cost. An alternative NAD(P)-dependent enzyme that does not generate hydrogen peroxide as a by-product, glycerol-3-phosphate dehydrogenase (G3PD), was therefore also investigated.

Three G3PD-encoding genes from *M*. *smegmatis* (G3PD_*Ms*_; YP885534, previously uncharacterised), *E*. *coli* (G3PD_*Ec*_; WP_001076194; [26]) *and Archaeoglobus fulgidus* (G3PD_*Af*_; WP_010878372; [23]) were expressed in *E*. *coli*, all of which produced soluble and active recombinant enzymes. The G3PD_*Ec*_ had been shown previously to be NAD-dependent [27], while G3PD_*Ms*_ (this study) and G3PD_*Af*_ require NADP [23]. To regenerate the nicotinamide cofactor, water-generating NADH oxidase from *Clostridium aminovalericum* (NOX_*Ca*_; accession no. BAE53714 [28]) and *Lactobacillus sanfransiscensis* (NOX_*Ls*_; accession no. BAB19268, PDB 2CDU; [29, 30]) were used; both were successfully produced in *E*. *coli*. NOX_*Ls*_ had been shown previously to regenerate both NAD^+^ and NADP^+^, albeit with a preference for NAD^+^ [29, 30].

The steady-state kinetics for each of the soluble enzymes were obtained (Table 1 & Supporting Information). The GlpKs had a *k*_cat_ range of 90–940 s^-1^ with *K*_M_ values of 15–350 μM for glycerol. GlpK_*Tk*_ was the most effective, with a *k*_cat_/*K*_M_ value of 6 x 10^7^ M^-1^.s^-1^, nearly three orders of magnitude higher than that of GlpK_*Cb*_ (Table 1). The AceK_Mt_ and PyrK_*Bs*_ had *k*_cat_/*K*_M_ values of 3 x 10^5^ M^-1^.s^-1^, while the AceK_*Ms*_ *k*_cat_/*K*_M_ value was approximately an order of magnitude higher at 3 x 10^6^ M^-1^.s^-1^ (Table 1).

The *k*_cat_/*K*_M_ values for the G3PDs did not vary greatly with a range of 1–7.5 x 10^5^ M^-1^.s^-1^. Of the NOXs, NOX_*Ls*_ was most efficient with *k*_cat_/*K*_M_ values of 0.9 and 2.9 x 10^7^ M^-1^.s^-1^ for NADH and NADPH, respectively, while NOX_*Ca*_ had a slightly lower second order rate constant of 0.5 x 10^7^ M^-1^.s^-1^ (for NADH only). GlpO_Mg_ had a *k*_cat_/*K*_M_ value that was approximately 10-fold higher than the most efficient G3PD (6.0 x 10^6^ M^-1^.s^-1^ *cf*. 7.5 x 10^5^ M^-1^.s^-1^) and GlpO_Ec_ (1.2 x 10^5^ M^-1^.s^-1^).

### Enzymatic cascades for DHAP production from glycerol

Both GlpK_*Tk*_ and GlpK_*Bs*_ were paired with AceK_*Mt*_ and AceK_*Ms*_ (Table 2) and the rates of G3P production were monitored. Enzyme loadings were normalised to *k*_*cat*_ values, as described in Methods and Materials, with consideration that cofactor-regeneration should always be the non-rate limiting reaction in order to drive the reaction towards increased product yield. Under the conditions tested, the GlpK_*Bs*_-AceK_*Mt*_ pair was the most active, with G3P production at 3.61 μM.s^-1^. The GlpK_*Tk*_-AceK_*Mt*_ pair produced G3P at 3.11 μM.s^-1^, while the paired enzymes that used AceK_*Ms*_ both had rates < 2 μM.s^-1^.

**Table 2:** Efficiency of selected heterogeneous paired reactions.

**Glycerol Kinase**^a^^,^^c^	**ATP Kinase**^b^^,^^c^	**Yield (%)**	**G3P Production Rate (μMs^-1^)**	**Turnover Number (ATP)**
**Pair 1: Glycerol kinase and acetate kinase pairs**				
GlpK_*Tk*_ (14.3; 56%)	AceK_*Mt*_ (11.3; 44%)	56.1 ± 3.2	3.11	56
GlpK_*Tk*_ (14.3; 42%)	AceK_*Ms*_ (20.1; 58%)	44.2 ± 1.3	2.24	44
GlpK_*Bs*_ (89.1; 89%)	AceK_*Mt*_ (11.3; 11%)	65.4 ± 4.3	3.61	65
GlpK_*Bs*_ (89.1; 82%)	AceK_*Ms*_ (20.1; 18%)	40.9 ± 3.1	2.27	41
**G3P dehydrogenase**	**NADH Oxidase**	**Yield (%)**	**DHAP Production Rate (μM s**^**-1**^**)**	**Turnover Number(NAD/NADP)**
G3PD_*Ec*_ (82.2; 89%)	NOX_*Ca*_ (10.1; 11%)	76.2 ± 5.4	4.20	76 (NAD^+^)
G3PD_*Af*_ (75.5; 74%)	NOX_*Ls*_ (27; 26%)	13.3 ± 1.5	0.72	13 (NADP)
G3PD_*Oc*_ (21; 68%)	NOX_*Ca*_ (10.1; 32%)	33.1 ± 2.5	1.83	33 (NAD^+^)
G3PD_*Ms*_ (82.2;75%)	NOX_*Ls*_ (27; 25%)	12.4 ± 1.0	0.67	12 (NADP)
GlpO_*Ec*_ (57.2; 100%)	n.a.	37.7 ± 0.6	2.09	n.a.
GlpO_*Mg*_ (85.6; 100%)	n.a.	62.5 ± 4.6	3.47	n.a.

^a,b^ Enzyme concentrations are given in parentheses in both pmoles per reaction and percentage (%) of total enzyme loading.

^c^ Reactions were conducted at room temperature in 1 mL total volume with 10 mM glycerol/glycerol-3-phosphate as starting substrate, 100 μM each of either ATP (Pair 1), NAD^+^ or NADP^+^ (Pair 2) and 10mM acetyl phosphate co-substrate (Pair 1, AceK). Samples were collected at various time points and analysed by LCMS. Values summarised in this table are based on 30 minute time point. Values represent the average of triplicate samples with standard deviation <10%.

n.a.–not applicable.

For the oxidation of G3P to DHAP two different approaches were tested- approach A using glycerophosphate flavoprotein oxidases (Fig 2A) and approach B using NAD(P)-dependent glycerol-3-phosphate dehydrogenases (Fig 2B). For approach A, GlpO_*Mg*_ was found to be catalytically superior to GlpO_*Ec*_, with a much enhanced *k*_cat_ and a superior *K*_M_, resulting in a tenfold difference in catalytic efficiency under similar molar enzyme loading conditions (Table 2). In approach B, G3PD_Ec_ was paired with NOX_*Ca*_ and G3PD_*Af*_ was paired with NOX_*Ls*_, reflecting their preferences for NAD and NADP, respectively. The G3PD_*Ec*_-NOX_*Ca*_ pair was considerably more efficient, with a DHAP production rate eight times greater than the G3PD_*Af*_-NOX_*Ls*_ pair (Table 2).

The paired enzymes were then coupled to constitute cascades that produce DHAP from glycerol. Eleven cascades were tested: nine utilizing G3PD-NOX enzyme combinations for conversion of glycerol-3-phosphate to DHAP and two using GlpO for this reaction in the cascade. The most active cascade utilising the G3PD-NOX enzyme combination was GlpK_*Bs*_-AceK_*Mt*_ and G3PD_*Ec*_-NOX_*Ca*_ with a DHAP production rate of 3.6 μM.s^-1^, albeit each of the cascades was reasonably active with DHAP production rates of > 1.6 μM.s^-1^ (Table 3). Despite some literature reports to the contrary for similar cascades coupling kinase enzymes with oxidase enzymes [7], we did not discover any inhibition of the glycerol kinase GlpK_*Tk*_ under the oxidative reaction conditions required for the four enzyme cascade, likely due to mitigation of the resultant peroxide by the addition of catalase. Although a simple rate of product formation after thirty minutes (μMs^-1^) is used for standardised comparative purposes in Tables 1–3, extra rate data (provided in S1 File, Part 3) illustrates that for all the final cascades, DHAP production rate decreases over time due to the inherent limitations of batch biocatalysis with enzymes which are product inhibited or operating in an equilibrium.

**Table 3:** DHAP production *via* two step enzymatic cascade.

Glycerol Kinase ^a^	ATP Kinase ^b^	G3P Dehydrogenase \ Oxidase ^c^	NADH Oxidase ^d^	% Total Conversion^e^	G3P Yield [mM] ^f^ (Production Rate [μM s^-1^])	DHAP Yield [mM] (Production [μM s^-1^])	ATP Turnover number (min^-1^)	NAD/NADP Turnover number (min^-1^)
GlpK_*Tk*_ (14.3; 12%)	AceK_*Mt*_ (11.3; 9%)	G3PD_*Ec*_ (82.2; 70%)	NOX_*Ca*_ (10.1; 9%)	44	4.41 (2.45)	1.89 (1.05)	44	19 (NAD)
GlpK_*Tk*_(14.3; 11%)	AceK_*Ms*_ (20.1; 16%)	G3PD_*Ec*_ (82.2; 65%)	NOX_*Ca*_ (10.1; 8%)	52	5.19 (2.88)	2.98 (1.66)	52	29 (NAD)
GlpK_*Tk*_ (14.3; 11%)	AceK_*Mt*_ (11.3; 9%)	G3PD_*Af*_ (75.5; 59%)	NOX_*Ls*_ (27; 21%)	41	4.09 (2.27)	1.00 (0.55)	41	10 (NADP)
GlpK_*Tk*_ (14.3; 10%)	AceK_*Ms*_ (20.1; 15%)	G3PD_*Af*_ (75.5; 55%)	NOX_*Ls*_ (27; 20%)	35	3.49 (1.94)	3.18 (1.77)	35	32 (NADP)
GlpK_*Tk*_ (14.3; 11%)	AceK_*Mt*_ (11.3; 8%)	G3PD_*Ms*_ (82.2; 61%)	NOX_*Ls*_ (27; 20%)	54	5.40 (3.00)	0.99 (0.55)	54	10 (NADP)
GlpK_*Bs*_ (89.1; 46%)	AceK_*Mt*_ (11.3; 6%)	G3PD_*Ec*_ (82.2; 43%)	NOX_*Ca*_ (10.1; 5%)	65	6.49 (3.61)	5.68 (3.16)	65	57 (NAD)
GlpK_*Bs*_ (89.1; 44%)	AceK_*Ms*_ (20.1; 10%)	G3PD_*Ec*_ (82.2; 41%)	NOX_*Ca*_ (10.1; 5%)	52	5.18 (2.88)	2.08 (1.16)	52	21 (NAD)
GlpK_*Bs*_ (89.1; 42%)	AceK_*Ms*_ (20.1; 8%)	G3PD_*Af*_ (75.5; 37%)	NOX_*Ls*_ (27; 13%)	41	4.08 (2.27)	3.29 (1.83)	41	33 (NADP)
GlpK_*Bs*_ (89.1; 44%)	AceK_*Mt*_ (11.3; 6%)	G3PD_*Af*_ (75.5; 37%)	NOX_*Ls*_ (27; 13%)	43	4.31 (2.41)	3.20 (1.77)	43	32 (NADP)
GlpK_*Tk*_ (14.3; 14%)	AceK_*Mt*_ (11.3; 11%)	GlpO_*Mg*_ (77.1; 75%)	-	63	6.30 (3.50)	4.19 (2.33)	63	NA
GlpK_*Tk*_ (14.3; 13%)	AceK_*Ms*_ (20.1; 18%)	GlpO_*Mg*_ (77.1; 69%)	-	88	8.89 (4.94)	7.38 (4.10)	88	NA
GlpK_*Bs*_ (89.1; 51%)	AceK_*Mt*_ (11.3; 6%)	GlpO_*Mg*_ (77.1; 43%)	-	62	6.20 (3.44)	2.30 (1.28)	62	NA

^a,b,c,d^ Enzyme concentrations are given in parentheses in both pmoles per reaction and percentage (molar %) of total enzyme loading. NA- not applicable.

^e^ Reactions were conducted at room temperature in 1 mL total volume with 10 mM glycerol as starting substrate, 100 μM of ATP, 100 μM NAD^+^ or NADP^+^ and 10mM acetyl phosphate co-substrate. Samples were collected at various time points and analysed by LCMS. Values summarised in this table are based on 30 minute time point and represent the average of triplicate samples, to the nearest second decimal place. Standard deviation was <10%. Total conversion represents the sum of glycerol-3-phosphate and DHAP product, expressed as a percentage of the starting glycerol substrate

^**f**^ Rate G3P production is based on the overall G3P produced, calculated from the combined yield of G3P and DHAP.

**Fig 3:**
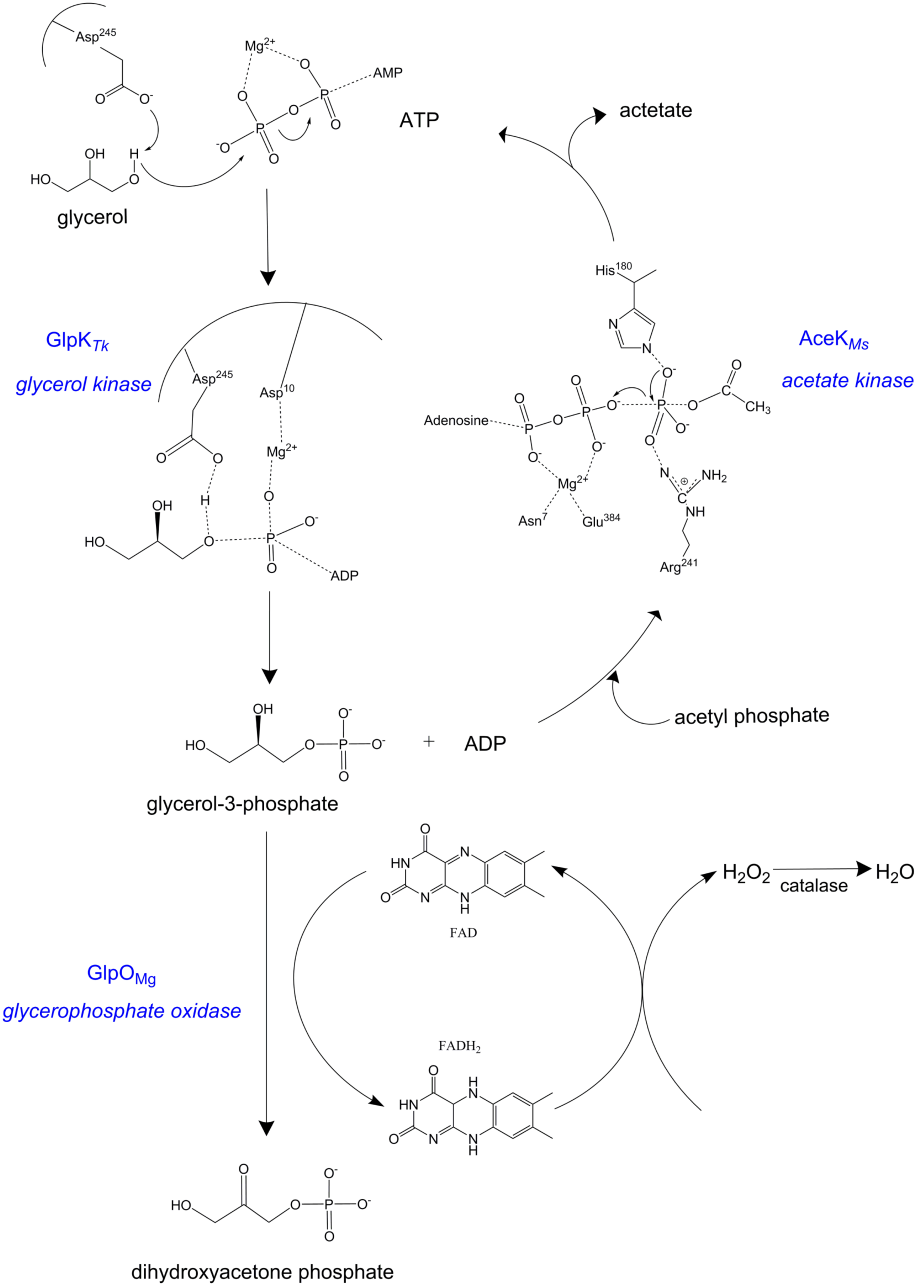
Details of the optimized cascade for the production of DHAP from glycerol. Phosphorylation of glycerol by ATP mediated by GlpK_*Tk*_ (EC 2.7.1.30) and Mg^2+^ *via* a phosphotransfer mechanism [33] was accompanied by regeneration of ATP from ADP by AceK_*Ms*_ (EC 2.7.2.1), which catalyzes reversibly the phosphorylation of acetate in the presence of a divalent cation and ATP with the formation of acetylphosphate and ADP[34]. Cytosolic glycerophosphate oxidase GlpO_*Mg*_(EC 1.1.3.21) likely converts glycerol-3-phosphate to DHAP by a similar mechanism to the related GlpO from *Mycoplasma pneumoniae* (4X9M) [35]), Similarly to other flavoprotein oxidases, glycerophosphate oxidase GlpO enzymes follow a hydride transfer mechanism to stabilize a positive charge on the flavin N(5)-sulfite adduct (C). The hydrogen peroxide generated from the oxidation of enzymatic FADH_2_ was converted to water by the addition of catalase from *Micrococcus lysodeikticus*.

Overall, the one-pot four-enzyme cascade comprising GlpK_*Tk*_-AceK_*Ms*,_ GlpO_*Mg*_ and catalase from *Micrococcus lysodeikticus* (Sigma 60634) was the most efficient of those tested in this study, producing 88% conversion of glycerol to DHAP in 60 mins at a rate of 4.1 μM.s^-1^ (Table 3, Fig 3), comparable with the most efficient systems described by Schumperli *et al*. [32]. Although a simple rate of product formation after thirty minutes (μMs^-1^) is used for standardised comparative purposes in Tables 1–3, extra rate data (provided in Supporting Information, Part 3) illustrates that for all the final cascades, DHAP production rate was initially fast and then decreased over time due to the inherent limitations of batch biocatalysis with enzymes which are product inhibited or operating in an equilibrium.

### Synthesis of chiral carbohydrates

**Fig 4:**
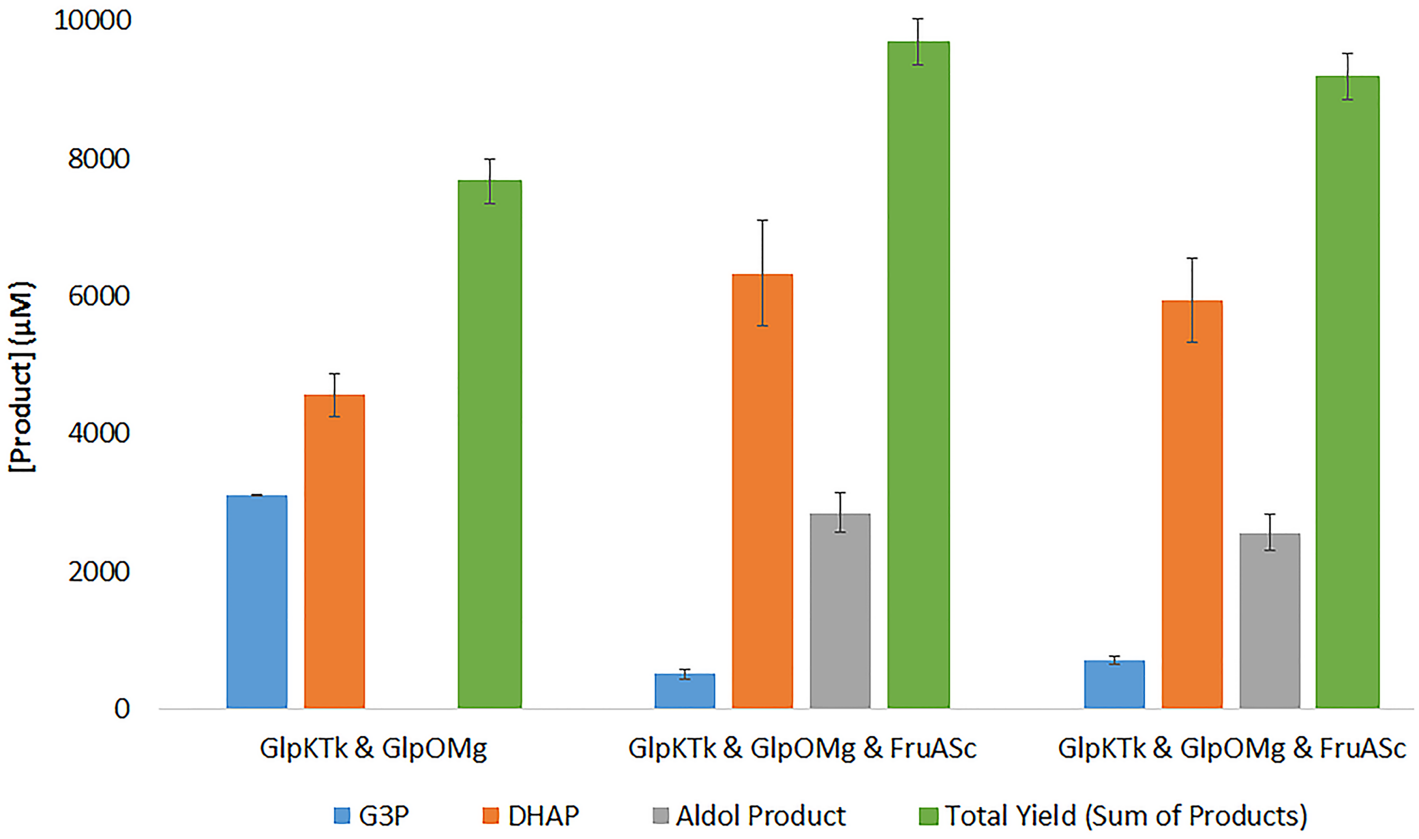
Production of rare chiral sugars by combining optimized multi-enzyme cascades for DHAP production with a DHAP-dependant fructose-1,6-biphosphate aldolase. Glycerol (10mM substrate) was converted to glycerol-3-phosphate by a glycerol kinase enzyme GlpK_*Tk*_ (28.6 pmoles) with concomitant regeneration of ATP by an acetate kinase enzyme AceK_*Ms*_ (40.2 pmoles). The glycerol-3-phopshate was then oxidized to DHAP by a novel *L*-glycerol-3-phosphate oxidase enzyme GlpO_*Mg*_ (154.2 pmoles), with mitigation of excess hydrogen peroxide by catalase (3U/mL) and an aldolase enzyme FruA_*Sc*_ (3.1 nmoles) converted this and acceptor aldehydes (provided at 10mM) into chiral sugars *D*-fructose-1,6-biphosphate (3*S*, 4*R*) and 3,4-dihydroxyhexulose phosphate (3*S*, 4*R*) as depicted.

The most efficient multi-enzyme cascade for the conversion of glycerol into DHAP, combining GlpK_*Tk*_, AceK_*Ms*_, GlpO_*Mg*_ and a commercial catalase enzyme for peroxide mitigation, was then coupled with a fructose-1,6-biphosphate aldolase enzyme from *Staphylococcus carnosus* (FruA_*Sc*;_ Uniprot Q07159; [36]) to demonstrate the production of chiral sugars using two different aldehyde acceptors (Fig 4).

The addition of the aldolase enzyme to the multi-enzyme cascade for DHAP production resulted in increased overall conversion of glycerol (Fig 4), likely due to a “pull-through” effect: as DHAP was consumed by the aldolase reaction, greater conversion to DHAP occurred due to mitigation of the product inhibition of GlpO_*Mg*_ by DHAP as reported previously [32]. This was consistent for both aldolase reactions tested.

The final selected quadruple enzyme cascade reaction was able to convert the simple, cheap starting material glycerol into dihydroxyacetone phosphate, introducing both a phospho- group and a reactive ketone. Additionally, coupling the DHAP-production enzyme cascade with an aldolase and a selected aldehyde, allowed the conversion of glycerol into chiral sugars *D*-fructose-1,6-biphosphate (3*S*, 4*R*) and 3,4-dihydroxyhexulose phosphate (3*S*, 4*R*) by a five-enzyme one-pot system, albeit without complete conversion to the aldol sugar.

Complete conversion to the final sugar in such a one-pot multi-enzyme batch reaction is unlikely to be feasible due to the product inhibition and equilibria constraints discussed above. Similar findings have been confirmed using an alternative *in vitro* multi-enzyme cascade for fructose-1,6-diphosphate synthesis from maltodextrin. This ATP-free four-step enzymatic synthesis, utilising alpha-glucan phosphorylase from *Thermotaga maritima*, phosphoglucomutase from *Thermococcus kodakarensis*, glucose 6-phosphate isomerase from *Thermus thermophilus*, and pyrophosphate phosphofructokinase from *T*. *maritima*, yielded 62.5% conversion of pyrophosphate to fructose-1,6-diphosphate over seven hours [37]. However, sophisticated algorithms are emerging to address the need for more accurate kinetic modelling of the optimal enzyme loading, reaction temperature, substrate loading, and cofactor concentration for *in vitro* multi-enzyme cascades [38]. When coupled with statistical comparisons such as global sensitivity analysis, global test comparisons or ensemble model robustness analysis [39], these can be used to pinpoint and optimize crucial bottlenecks in multi-enzyme cascade reactions. In the example listed above, experimental optimisation of enzyme loading ratios and step-wise substrate addition resulted in an improved yield per time of fructose-1,6-diphosphate for the multi-enzyme cascade [37]. Further optimization of the multi-enzyme cascade described herein should be achievable through application of these methods.

Additionally, the rapid initial rate of DHAP production achieved intially by some of the multi-enzyme cascades described here (see supporting information Part 3) might be maintained more effectively throughout the reaction time by utilising enzyme immobilization and continuous flow catalysis to enhance biocatalytic productivity [40].

Thus for commercial applications, engineering non-limited enzyme catalysts, applying a continuous flow enzyme cascade system or balancing the space time yield of the cascade reaction versus complete conversion and final product purity would likely be necessary to complete the optimization of the multi-enzyme cascade for each desired application. Nonetheless, the intractable nature of rare chiral sugar synthesis by chemocatalysis makes multi-enzymatic cascades that are capable of introducing two new stereocentres in the final molecule an attractive prospect for biobased manufacturing applications.

## Conclusion

We have examined and optimized the conversion of the cheap, readily available substrate glycerol into sugar analogs by one-pot, multi-enzyme cascade reactions focusing on the most efficient conversion of glycerol to DHAP by rate rather than yield.

In addition, we have characterized the enzyme activity of sixteen enzymes, including four novel enzymes involved in the reaction cascades, one for each of the four classes of enzymes employed to convert glycerol to DHAP (Table 1). During this process we have also identified a novel glycerophosphate oxidase enzyme from a *Mycoplasma* with superior catalytic efficiency to previously reported glycerophosphate oxidase enzymes.

## Supporting information

S1 Table Summary of the recombinant expression and purification of candidate enzymes. (DOCX)

S1 File Comprises: Construction of expression vectors: Cloning and Expression of Genes Encoding Enzymes of Interest (1); Kinetic Data: Supplementary Information (2); Example of the variance in overall production rate (3). (DOCX)
