# Supplementary material for: Sugar analog synthesis by in vitro biocatalytic cascade: A comparison of alternative enzyme complements for dihydroxyacetone phosphate production as a precursor to rare chiral sugar synthesis
Source: PLoS One. 2017 Nov 7;12(11):e0184183. doi: 10.1371/journal.pone.0184183 (PMC5675407; doi:10.1371/journal.pone.0184183)
Supplement: S1 Table — (DOCX) [file pone.0184183.s001.docx]

**Supplementary Table 1. Summary of the Recombinant Expression and Purification of Candidate Enzymes**

| **Enzyme** | **Source** | **Accession Number**  **(PDB No.)** | **Size (kDa)** | ***E.coli* Expression Host Cells** | **Expressed** | **Protein Yield*** |
| --- | --- | --- | --- | --- | --- | --- |
|  |  |  |  |  |  |  |
| **Glycerol** | *Escherichia coli* | CQR83322 | 56.226 | BL21DE3* | low expression | n.d. |
| **Kinase** | *Clostridium beijerinckii* | AIU00287 | 55.500 | BL21AI | yes | 20 |
|  | *Mycobacterium smegmatis 6229* | AFP42499 | 55.890 | BL21DE3* | yes | n.d. |
|  | *Mycobacterium smegmatis 6756* | YP890964 | 55.094 | BL21DE3* | low expression | n.d. |
|  | *Thermococcus kodakarenis* | O93623 (2ZF5) | 55.890 | BL21DE3* | yes | 20 |
|  | *Saccharomyces cerevisiae* | P32190 | 79.890 | BL21AI | low expression | n.d. |
|  |  |  |  | BL21DE3* | low expression | n.d. |
|  | *Haloferax volcanii* | D4GYI5 | 56.733 | BL21DE3* | low expression | n.d. |
|  | *Bacillus stearothermophilus* | Sigma G0774 |  | n/a | n/a |  |
| **Acetate Kinase (AK)** | *Mycobacterium smegmatis 0784* | WI_011727188 (4IJN) | 40.200 | BL21AI | yes | 10 |
|  | *Methanosarcina thermopila* | WP_048129005 (1TUU) | 44.400 | BL21AI | yes | n.d. |
|  |  |  |  | BL21DE3* | yes | 1 |
| **Pyruvate Kinase (PK)** | *Bacillus stearothermophilus* | WP_033014443 (2E28) | 63.490 |  | low expression | n.d. |
| **Glycerol-3-Phosphate** | *Mycobacterium smegmatis 1140* | YP885534 | 36.390 | BL21AI | yes | 10 |
| **Dehydrogenase** | *Mycobacterium smegmatis 2393* | YP886733 | 33.669 | BL21AI | low expression | n.d. |
|  | *Escherichia coli* | WP_001076194 | 36.470 | BL21DE3* | yes | 30 |
|  | *Clostridium beijerinckii 1135* | A6LSI7 | 35.914 | BL21DE3* | low expression | n.d. |
|  | *Saccharomyces cerevisiae* | Q00055 | 42.877 | BL21DE3* | insoluble | n.a. |
|  | *Archaeglobus fulgides* | WP_010878372 | 36.894 | BL21DE3* | yes | 10 |
|  | *Oryctolagus cuniculus* (muscle) | Sigma G6880 |  | Not applicable | |  |
| **NOX** | *Lactococcus lactis* | A2RIB7 | 48.980 | BL21DE3* | yes | 1 |
|  | *Clostridium aminoverlaricum* | BAE53714 | 49.400 | BL21DE3* | yes | 1 |
|  | *Streptococcus mutans* | [Q54453](javascript:Sequence2('Q54453')) | 50.020 | BL21DE3* | low expression | n.d. |
|  | *Lactobacillus sanfrancescensis* | BAB19268 | 49.646 | BL21DE3* | yes | 1 |
| **GlpO** | *Enterobacteriaceae casseliflavus* | WP_005237472 | 69.374 | BL21DE3* | yes | 15 |
|  | *Mycoplasma gallisepticum* | WP_014574362 | 46.680 | BL21AI | yes | 20 |
| **catalase** | *Micrococcus lysodeikticus* | Sigma 60634 |  | Not applicable | |  |
| **FruA*_Sc_*** | *Staphylococcus carnosus* | Q07159.3 | 32.849 | BL21DE3* | yes | 5-10 |

* yield - mg protein L^-1^ culture; n.d. – not determined; n.a. – not applicatble
