# Supplementary material for: Sugar analog synthesis by in vitro biocatalytic cascade: A comparison of alternative enzyme complements for dihydroxyacetone phosphate production as a precursor to rare chiral sugar synthesis
Source: PLoS One. 2017 Nov 7;12(11):e0184183. doi: 10.1371/journal.pone.0184183 (PMC5675407; doi:10.1371/journal.pone.0184183)
Supplement: S1 File — (DOCX) [file pone.0184183.s002.docx]

**Supporting Information**

1. **Construction of expression vectors: Cloning and Expression of Genes Encoding Enzymes of Interest**

Supplementary information about the enzymes assessed in this research is included in Supplementary Table 1. Additionally, the sequences listed below provide the exact DNA sequence utilised for the construction of expression vectors for each protein. Supplementary Figure 1 shows a map of the expression vectors used and indicative cloning sites. Plasmids used in enzyme purification were constructed either on the pETCC2 (this study) or pDEST17 (Invitrogen, Thermo-Fisher Scientific) vectors, which contained a Histidine (His) tag, isopropyl β-D-1-thiogalactopyranoside–inducible promoter, and high copy origin of replication. The plasmids were maintained with 100 mg/L ampicillin.

**Supplementary Figure 1.** Expression vectors used in this study. Constructs were cloned into pETCC2::eGFP by *Nde*I-*Bam*HI sites, replacing the indicator eGFP gene, or into pDEST17 by Gateway cloning procedures from a pDONR vector, as per manufacturer’s instructions (Invitrogen, ThermoFisher Scientific, USA), replacing the indicator chloramphenicol acyl transferase (CmR) gene. All constructs were confirmed by sequencing across the entire insert (Macrogen, South Korea).

**DNA Coding Sequences Used In This Study.**

**Glycerol kinases:**

**GlpK*_Tk_*** (restriction enzyme cloning sites in bold; pETCC2)

**catatg**gaaaaatttgtgctgagcctggatgaaggtacaaccagcgcacgtgcaattatctttgatcgtgaaagcaacattcatggcattggccagtatgaatttccgcagcattatccgcgtccgggttgggttgaacataatccggaagaaatttgggatgcacagctgcgtgccattaaagatgcaattcagagcgcacgtattgaaccgaatcagattgcagcaattggtgttaccaatcagcgtgaaaccaccctggtttgggataaagatggtaaaccgctgtataatgcaattgtttggcagtgtcgtcgtaccgcagaaatggttgaagaaattaaacgtgaatacggcaccatgatcaaagaaaaaaccggtctggttccggatgcatattttagcgcaagcaaactgaaatggctgctggataatgttccgggtctgcgtgaaaaagccgaaaaaggtgaagttatgtttggcaccgttgatacctttctgatttatcgtctgaccggtgaacatgtgaccgattatagcaatgcaagccgtaccatgctgtttaacatcaaaaaactggattgggatgatgagctgctggaactgtttgatattccggaaagcgttctgccggaagttcgtgaaagtagcgaagtttatggctataccaaaaaagaactgctgggtgcagaaattccggttagcggtgatgccggtgatcagcaggcagcactgtttggtcaggcagcatttgaagcaggtatggttaaagcaacctatggcaccggtagctttattctggttaataccgataaaatggtcctgtatagcgataatctgctgaccaccattgcatggggtctgaatggtcgtgttagctatgcactggaaggtagcatttttgttaccggtgcagcagttcagtggctgcgtgatggcattaaaatcattaaacatgccagcgaaaccgaagaactggcaaccaaactggaaagcaatgaaggtgtttattttgtgcctgcatttgttggtctgggtgcaccgtattgggatcagtttgcacgtggtattatcattggtattacccgtggtacaggtcgtgaacatctggcacgtgcgaccctggaagcaattgcatatctgacccgtgatgttgttgatgagatggaaaaactggtgcagattaaagaactgcgtgttgatggtggtgcaaccgcaaatgattttctgatgcagtttcaggccgatattctgaatcgtaaagttattcgtccggtggtgaaagaaaccaccgcactgggtgccgcatatctggcaggtctggcagttgattattgggcagatacccgtgaaattgcagaactgtggaaagcagaacgtatttttgagccgaaaatggatgaaaaaacccgtgaacgtctgtacaaaggttggaaagaagcagttaaacgcgcaatgggttgggcaaaagttgttgatagcgcaaaaagcaac**ggatcc**

**GlpK*_Hv_*:** ((restriction enzyme cloning sites in bold; pETCC2)

**catatg**agcggtgaaacctatgttggtgcaattgatcagggcaccaccggcacccgttttatggtttttgatcatgatggtaaagttgtggccaacgcctatgaaaaacatgagcagatttatccggaaccgggttgggttgaacatgatgcaaatgaaatttgggacaacaccaaacaggttattgatgcagcactgagcagcgcaggtctggatgcagaacagctggaagcaattggcattaccaatcagcgtgaaaccaccctggtttgggatcgtgaaaccggtcagccgattcataatgcaattgtttggcaggatcgtcgtaccaccgatcgtattgaaaccctggaagccgaaggtaaaaccgatgatgttcgtgcaaaaaccggtctggaaccggatgcatattttagcgcaaccaaagcagaatggctgctggataatagcgatccgattaaactgcagcgtagccgtccggaagatattcgtgatcgtgcagcagatggtgaactggcatttggcaccattgatacctggctgatttataacctgaccggcaatcatattaccgatgttaccaatgcaagccgtaccatgctgtttaacattcatgatatggaatgggatgatgaactgctggatgaatttaatgttcctcgcgaactgctgccggaagttcgtccgagcagtgatgatgattattatggtacaaccgatgccgatggttttctgggtgccgaagttccggttgccggtgcactgggtgatcagcaggcagcactgtttggtcagacctgttttgatgccggtgatgcaaaaaatacctatggcaccggtagctttatgctgatgaataccggtgatgaagcagttatgagcgaacatggtctgctgaccaccgttggttttcagcgtagcggtgaaccggtgcagtatgcactggaaggtagcatttttatcaccggtgccgcaattgaatggctggaagatatgaccctgattgataacgcagcagaaagcgaaaaactggcacgtagcgttgaaagcaccgatggtgtttattttgttccggcatttacgggtctgggtgcaccgcattgggatcagcgtgcacgtggtacaattgttggtatgacccgtggcacccgtcgtgaacatattgttcgtgccaccctggaaagcattgcatttcagacccgtgatgttgcagaagcaatggaaagcgatagcgaaattgatctgagcagcctgcgtgttgatggtggtgcagttaaaaacaattttctgtgtcagctgcagagcaacattctggataccgaaattgttcgtccgcaggttgatgaaaccacagccctgggtgcagcctatgcagccggtctggcagttggttattgggaaacactggatgagctgcgtgaaaattggcaggtagatcgtgaatttgcaccgaaagatccgcagaatgtggaacatcgttatggtcgttggaaagaagcagttgatcgtagcctggattgggcacgtgaagaatagta**ggatcc**

**GlpK*_Ec_*** (restriction enzyme cloning sites in bold; pETCC2)

**catatg**agcggtgaaacctatgttggtgcaattgatcagggcaccaccggcacccgttttatggtttttgatcatgatggtaaagttgtggccaacgcctatgaaaaacatgagcagatttatccggaaccgggttgggttgaacatgatgcaaatgaaatttgggacaacaccaaacaggttattgatgcagcactgagcagcgcaggtctggatgcagaacagctggaagcaattggcattaccaatcagcgtgaaaccaccctggtttgggatcgtgaaaccggtcagccgattcataatgcaattgtttggcaggatcgtcgtaccaccgatcgtattgaaaccctggaagccgaaggtaaaaccgatgatgttcgtgcaaaaaccggtctggaaccggatgcatattttagcgcaaccaaagcagaatggctgctggataatagcgatccgattaaactgcagcgtagccgtccggaagatattcgtgatcgtgcagcagatggtgaactggcatttggcaccattgatacctggctgatttataacctgaccggcaatcatattaccgatgttaccaatgcaagccgtaccatgctgtttaacattcatgatatggaatgggatgatgaactgctggatgaatttaatgttcctcgcgaactgctgccggaagttcgtccgagcagtgatgatgattattatggtacaaccgatgccgatggttttctgggtgccgaagttccggttgccggtgcactgggtgatcagcaggcagcactgtttggtcagacctgttttgatgccggtgatgcaaaaaatacctatggcaccggtagctttatgctgatgaataccggtgatgaagcagttatgagcgaacatggtctgctgaccaccgttggttttcagcgtagcggtgaaccggtgcagtatgcactggaaggtagcatttttatcaccggtgccgcaattgaatggctggaagatatgaccctgattgataacgcagcagaaagcgaaaaactggcacgtagcgttgaaagcaccgatggtgtttattttgttccggcatttacgggtctgggtgcaccgcattgggatcagcgtgcacgtggtacaattgttggtatgacccgtggcacccgtcgtgaacatattgttcgtgccaccctggaaagcattgcatttcagacccgtgatgttgcagaagcaatggaaagcgatagcgaaattgatctgagcagcctgcgtgttgatggtggtgcagttaaaaacaattttctgtgtcagctgcagagcaacattctggataccgaaattgttcgtccgcaggttgatgaaaccacagccctgggtgcagcctatgcagccggtctggcagttggttattgggaaacactggatgagctgcgtgaaaattggcaggtagatcgtgaatttgcaccgaaagatccgcagaatgtggaacatcgttatggtcgttggaaagaagcagttgatcgtagcctggattgggcacgtgaagaatagta**ggatcc**

**GlpK*_Ms_*_6229_** (restriction enzyme cloning sites in bold; pETCC2)

**catatg**gccgatttcgtcgcagcgatcgaccagggcaccacgagcacgcggtgcatgatcttcgaccatgacggcgccgaagtgggccgccaccagctcgaacacgagcagatcctgcccagggcgggctgggtcgagcacaacccggtggagatctgggagcgcaccgcatcggtggtgatgaccgcactcaaccggacgaatctgcaggcctccgacctggccgcgctgggcatcaccaaccagcgcgagacgtcgctggtgtggaaccggcacaccggcaggccctactacaacgcgatcgtgtggcaggacacccgcaccgacagcatcgccgcggcactcgaccgtgacggccgcggtgacgtcatccggcgcaaggccggtctgccgcccgcgacgtacttctcgggcggcaagatccagtggattctggacaacgtgccgggggtccgcgaggacgccgagaagggcgacgcgatcttcgggaccgccgacagttggctggtgtggaacctcaccggcggtacgcgcggcggcgtgcacgtcaccgacgtcaccaacgccagccgcaccatgctgatgaacctggagaccctcgactgggacgacgaactgttgtcgttcttcgggattccgcgccggatgctgcccgagatcaagccgtcgtcgtatccggagtcgtacggcatcacgcgcgacgacggtccgctggccgggcaggtgccgttgaccggcatcctcggtgaccagcaggccgcgatggtcggccaggtgtgcctggaggccggcgaggccaagaacacctatggcaccggcaatttcctgctgctcaacaccggtgagaagatcgtgcgctcggacaacggtctgctgaccacggtgtgctaccagttcggcgactcgaaaccggtttacgcgcttgagggttcgatcgcggtgaccggatcggccgtgcagtggttgcgcgaccagttgggcatcatcagcggtgcgtcgcagagcgagtcgctggcgcggcaggtcagcgacaacggcggcgtgtacttcgtgcccgccttctccggcctgttcgcgccgtactggcgcagcgacgcgcgcggcgcgatcgtgggcctgtcgcggttcaacaccaacgcgcacctggcgcgtgcgacgctggagtcgatctgctaccagagccgcgacgtggtcgacgccatggaggcggattccggtgtgcaccttgaggttctcaaggtcgacggtgggatcaccgccaacgcgctgtgcatgcagatccaggcggacgtgctcggcgtcgacgtggtcaagcccctggtcgccgagaccaccgcgctcggtgcggcgtacgcggccggtctggcggtgggcttctgggagaacgccgacgacttgcgggccaactggcaggaggacaagcgctggtcaccgcagtggtcagatgagcaacgcgagaagggatacgcgggctggcagaaggccgttcagcgcaccctcgactgggtcgagatcgagtga**ggatcc**

**GlpK*_Ms_*_6756_** ((restriction enzyme cloning sites in bold; pETCC2))

**catatg**accgagcgctacatcgccgcgctcgaccagggaacgacctccacccgctgcatcgtctacgaccaccacggcaggctggtctcggtcgcgcagcgcgaacaccgccagtactacccgcggccgggctgggtcgagcacgacccgaccgagatctgggagatcgtgcgccggatcatcccgcaggccctcgccgacgccgacgtcgaaccgcgccagatcatggcgctcggcatcaccaaccagcgcgagaccaccgtcgtgtgggaccgccacaccggcaagcccgcgcaccgcgcgatcgtgtggcaggacaaccggaccgcgggtctgctgccggccatcgcggccgaactcgacgacgagattttgctcgcgcgttgcggtctgccgctcgcgacgtacttctccggaccgaagctgcgttggctgttggacaacgatcgtgacctgcgcgcccgcgccgagagcggggatctgctgttcggcaccgtcgattcgtggttgctgtggaacctcaccggcggcatcgacggcggcgtgcacgccaccgacgtcaccaacgccagccgcaccatgctgatgaacctggagaccctcgactgggacgacgaactgctcgcgggcatgcacgtaccgcgacgcatgatgcccgagatccggcccaccaacgggccgttcccgtcgaccgtcgaccccatcgcgggcatcccgatcaccgcggtcatcggtgatcagcaggcatccctgttcggccagacggcattcgaggccggcgaggcaaagtgcacgttcggcaccggaagcttcgtcctgctcaacaccggcaaggacatcgtgcggtcgcagcgtgggctgctcaccaccgtcgcccgcaagttcgacggtgagcccgcggtgtacgcgctggagggatcggttgccgtggccggtgcgctggtcgcgtggtcgcgcgacaacctcggcctggtgaagacgccggccgagatcgaaacgctcgcgcgcacagtcgaagacaacggcggctgctacatcgtgccggccttctcgggcctgtactcgccgtactgggccaccgaggcgcgcggtctggtggtcgggctgacctcctatgtcaccaaggggcacatcgccagagcggtcctggaagccaccgcatggcagatccgcgacgtcatcgacgcgatgaaagacgactccgacatcgccgtgcgctcactgcacgtcgacggcggtatgacggccgacaacctgctcatgcaactcgtcgccgacattctcgacgcgcccgtcgtgcggcccatgatggccgaaaccgtcgctctcggagcggcttacgcagccggtctggcggcgggctactggcccgaccgtgcggtgctgcggtccaactggcgccgcgcggccgagtggcggcccagcatggacccgacccgccgccaacgtgaactcgacgactggcaccgcgcggtgcaattggcgatcagttggggcgacggacgccgcgaaaccgtgtga**ggatcc**

**Acetate Kinase Enzymes:**

**AceK*_Ms_*** (Gateway cloning sites in bold; into pDEST17)

**acaagtttgtacaaaaaagcaggcttc**catatgaccgttctggttgttaatagcggtagcagcagcctgaaatatgcagttgttcgtccggcaagcggtgaatttctggcagatggtattattgaagaaattggtagcggtgcagttccggatcatgatgcagcactgcgtgcagcatttgatgaactggcagcagcaggtctgcatctggaagatctggatctgaaagcagttggtcatcgtatggttcatggtggtaaaaccttctataaaccgagcgttgttgatgatgagctgattgcaaaagcacgtgaactgagtccgctggcaccgctgcataatccgcctgcaattaaaggtattgaagttgcacgtaaactgctgccggatctgccgcatattgcagtttttgataccgccttttttcatgatctgcctgcaccggcaagcacctatgcaattgatcgtgaactggccgaaacctggcatattaaacgttatggttttcatggcaccagccatgaatatgttagccagcaggcagcaatttttctggatcgtccgctggaaagcctgaatcagattgttctgcacctgggtaatggtgcaagcgcaagcgcagttgccggtggtaaagccgttgataccagcatgggtctgaccccgatggaaggtctggttatgggcacccgtagcggtgatattgatccgggtgttattatgtatctgtggcgtaccgcaggtatgagcgttgatgatattgaaagcatgctgaatcgtcgtagtggtgttctgggtctgggtggtgcgagcgattttcgcaaactgcgcgaactgattgaaagcggtgatgaacatgcaaaactggcctatgatgtgtatattcatcgtctgcgcaaatatatcggtgcatatatggcagtgctgggtcgtaccgatgttattagctttaccgcaggcgttggtgaaaatgttccgcctgttcgtcgtgatgccctggcaggcctgggtggtctgggtattgaaattgatgatgcactgaatagcgcaaaaagtgatgaaccgcgtctgattagcacaccggatagccgtgttacagttctggtggttccgaccaatgaagaactggcaattgcacgtgcctgtgttggtgttgtttaagaattcg**acccagctttcttgtacaaagtggt**

**AceK*_Mt_*** (restriction enzyme cloning sites in bold; pETCC2)

**catatg**aaagtgctggttattaatgcaggtagcagcagcctgaaataccagctgattgatatgaccaatgaaagcgcactggcagttggtctgtgtgaacgtattggtattgataacagcatcatcacccagaaaaaattcgacggcaaaaaactggaaaaactgaccgatctgccgacccataaagatgcactggaagaagttgttaaagccctgaccgatgatgaatttggcgtgattaaagatatgggcgaaattaatgccgttggtcatcgtgttgttcatggtggtgaaaaattcaccacctcagcactgtatgatgaaggtgttgaaaaagccatcaaagattgctttgaactggcaccgctgcataatccgcctaatatgatgggtattagcgcatgtgcagaaatcatgcctggcaccccgatggttattgtttttgataccgcatttcatcagaccatgcctccgtatgcatatatgtatgcactgccgtatgatctgtatgaaaaacatggtgtgcgcaaatatggttttcatggcaccagccataaatatgttgcagaacgtgcagcactgatgctgggtaaaccggcagaagaaaccaaaatcattacctgtcatctgggtaatggtagcagtattaccgcagttgaaggtggtaaaagcgttgaaaccagcatgggttttacaccgctggaaggtctggcaatgggcacccgttgtggtagcattgatccggcaattgttccgtttctgatggaaaaagaaggtctgaccacacgcgaaattgataccctgatgaacaaaaaaagcggtgttctgggtgttagcggtctgagcaatgattttcgtgatctggatgaagcagcaagcaaaggtaatcgtaaagcagaactggccctggaaatttttgcctataaagtcaaaaaattcatcggcgagtatagcgcagttctgaatggtgcagatgcagttgtttttaccgcaggtattggtgaaaatagcgcaagcattcgtaaacgtattctgaccggtctggatggtattggcattaaaatcgatgatgagaaaaacaaaatccgtggccaagaaatcgatattagcacaccggatgcaaaagttcgtgtttttgttattccgaccaacgaagaactggcaattgcacgtgaaaccaaagaaattgttgaaaccgaagttaaactgcgtagcagcattccggtttaa**ggatcc**

**PyrK*_Bs_*** (restriction enzyme cloning sites in bold; pETCC2)

**catatg**aaacgcaaaaccaaaattgttagcaccattggtccggcaagcgaaagcgttgataaactggttcagctgatggaagcaggtatgaatgttgcacgtctgaattttagccatggcgatcatgaagaacatggtcgtcgtattgcaaatattcgtgaagcagcaaaacgtaccggtcgtaccgttgcaattctgctggataccaaaggtccggaaattcgtacccataatatggaaaatggtgccatcgaactgaaagaaggtagcaaactggttattagcatgagcgaagttctgggcacaccggaaaaaatcagcgttacctatccgagcctgattgatgatgttagcgttggtgcaaaaatcctgctggatgatggtctgattagcctggaagttaatgcagtggataaacaggcaggcgaaattgtgaccaccgttctgaatggtggtgttctgaaaaacaaaaaaggtgtgaatgttccgggtgtgaaagttaatctgcctggtattaccgaaaaagatcgtgccgatattctgtttggtattcgtcagggcattgattttattgcagcaagctttgttcgtcgtgcaagtgatgttctggaaattcgcgaactgctggaagcacatgatgcactgcatattcagattatcgccaaaatcgaaaatgaagaaggcgtggccaacattgatgaaattctggaagcagcagatggcctgatggttgcacgtggtgatctgggtgttgaaattccggcagaagaagttccgctgattcagaaactgctgatcaaaaaaagcaacatgctgggtaaaccggttattaccgcaacccagatgctggatagcatgcagcgtaatccgcgtccgacccgtgccgaagcatcagatgttgcaaatgcaatttttgatggcaccgatgcagttatgctgagcggtgaaaccgcagcaggtcagtatccggttgaagcagttaaaaccatgcatcagattgcactgcgtaccgaacaggcactggaacatcgtgatattctgagccagcgtaccaaagaaagccagaccaccattaccgatgcaattggtcagagcgttgcacataccgcactgaatctggatgttgcagcaattgttaccccgaccgttagcggtaaaacaccgcagatggttgccaaatatcgtccgaaagcaccgattattgcagttaccagcaatgaagccgttagccgtcgtctggcactggtttggggtgtttatacgaaagaagcaccgcacgttaataccaccgacgaaatgctggatgtggcagttgatgcagccgttcgtagcggtctggttaaacatggtgacctggttgttattacagccggtgttccggttggcgaaaccggtagcacaaatctgatgaaagttcatgttattagcgacctgctggcaaaaggtcagggtattggtcgtaaaagcgcatttggtaaagcagttgttgcaaaaaccgcagaagaggcacgtcagaaaatggttgatggtggcattctggttaccgttagtaccgatgccgacatgatgccagcaattgaaaaagcagcagccattattacggaagaaggtggtctgaccagccatgcagcggttgttggtctgagcctgggtattccggtgattgttggtgtggaaaatgcaaccaccctgtttaaagatggtcaagaaattaccgtggatggtggttttggtgcagtttatcgtggtcatgcaagcgttct**ggatcc**

**Glycerol-3-Phosphate Dehydrogenase Enzymes:**

**G3PD*_Ms1140_*** (Gateway cloning sites in bold; into pDEST17)

**acaagtttgtacaaaaaagcaggcttc**gcagcaagcctgcgtgttccgaaagttgttgttctgggtggtggtagctggggcaccaccgttgcaagcatttgtgcacgtcgtggtccgaccctgcagtgggttcgtagcgaagaaaccgcaaaagatattaatgaaaatcaccgcaatagccgctatctgggtgatgatgttgtgctgccggatagcctgaccgcaaccaatgattttagcgaagcagcagcatgtgcagatgttattgttatgggtgttccgagccatggttttcgtggtgttctgcaagaactggcacgtgaactgcgtccgtgggttccggttgttagcctggttaaaggtctggaacagggcaccaatctgcgtatgagccagattgttgatgaagttctgcctggtcatccggcaggtattctggcaggtccgaatattgcacgtgaagttgccgaaggttatgcagcagcagccgttctggcaatgccggatcagcatctggcagcaaaactggcagaactgtttcgtaccaaacgttttcgtacctataccaccgatgatgttaccggtgttgaaatggcaggcgcactgaaaaatgtttatgcaattgccgttggtatgggttatagcctgggtattggtgaaaatacccgtgcaatggttatggcacgtgcagttcgtgaaatggccaaactgggtgaagcagccggtggtcatcgtgatacctttgcaggtctggcaggcatgggtgatctgattgttacctgtaccagccagcgtagccgtaatcgtcatgttggtgaacagctgggtgcaggcaaaaaaatcgatgaaattattgccagcatgaaccaggttgcagaaggtgttaaagcagcaagcgttattatggaatttgccgatcagtatggtctgaatatgccgatcgcacgcgaagttgatgcagttattaatcatggtagcagcgttgaacaggcatatcgtggtctgatggcagaaaaaaccggtcatgaagttcatagcagcggcttttaaggatcc**acccagctttcttgtacaaagtggt**

**G3PD*_Ms2393_*** (Gateway cloning sites in bold; into pDEST17)

**acaagtttgtacaaaaaagcaggcttc**gaagcagcagttatgggttgtggtgcatggggcaccgcactggcaaaagttctggcagatgcaggtaatccggttaccatgtgggcacgtcgtccggaagttgcagatgaaattaacagcgaacatcgtaatagcgagtatctgggtgatgttgttctgcctgcaagcattcgtgcaaccagcgatccggaagaggcactgcgtggtgcctgtaccgttatgctggcagttccgagccagaaactgcgtgcaaatctggataattggaaacacctgattgaagatgatgttaccctggttagcctggccaaaggtattgaactgggcaccctgatgcgtatgagccaggttattgttcaggttaccggtgcagatccgagccgtgttggtgttgttagcggtccgaatctggcaagcgaaattgccgatgaacagcctgcagcaaccgttattgcatgtagcgatagcggtcgtgcagttaccctgcagcgtgcaatggcaaccggttattttcgtccgtataccaatccggatgttattggtgccgaagttggtggtgcgtgtaaaaatgttattgcactggcatgtggtatggcagttggtgtgggtctgggtgaaaataccgttgcagcactgattacccgtggtctggcagaaattatgcgtctgggtattgccctgggtgcaacaccggcaacactggctggcctggcaggcgttggtgatctggttgccacctgtaccagtccgcatagccgtaatcgtacctttggtgaacgcctgggtaaaggtggcaccatggaatcagcactggcagcagccggtggtcatgttgccgaaggtgttgcaagctgtgaaagcgttctggccctggcaagcagctatggtgttgaaatgccgctgaccgatgcagttcatcgtgtttgtcataaaggtctgagcgttcatgaagcagttgcactgctgctgggtcgtagcaccaaaccggaataaggatcc**acccagctttcttgtacaaagtggt**

**G3PD*_Ec_*** (restriction enzyme cloning sites in bold; pETCC2)

**catatg**aatcagcgtaatgcaagcatgaccgttattggtgcaggtagctatggcaccgcactggcaattaccctggcacgtaatggtcatgaagttgttctgtggggtcatgatccggaacacattgcaaccctggaacgtgatcgttgtaatgcagcatttctgccggatgttccgtttccggataccctgcatctggaaagcgatctggcaacagcactggcagcaagccgtaatattctggttgttgttccgagccatgtttttggtgaagttctgcgtcagattaaaccgctgatgcgtccggatgcacgtctggtttgggcaaccaaaggtctggaagcagaaaccggtcgtctgctgcaggatgttgcacgtgaagcactgggtgatcagattccgctggcagttattagcggtccgacctttgcaaaagaactggcagccggtctgccgaccgccattagcctggcaagcaccgatcagacatttgccgatgatctgcagcaactgctgcattgtggtaaaagctttcgtgtttatagcaacccggattttattggtgttcagctgggtggtgcagtgaaaaatgttattgcaattggtgccggtatgagtgatggtattggttttggtgcaaatgcacgtaccgcactgattacccgtggtctggccgaaatgagccgtctgggtgcagccctgggtgccgatccggcaacctttatgggtatggcaggtctgggcgatctggttctgacctgtaccgataatcagagccgtaatcgtcgttttggtatgatgctgggtcagggtatggatgttcagagcgcacaagaaaaaattggtcaggttgttgaaggttaccgcaataccaaagaagtgcgcgaactggcacatcgttttggcgttgaaatgccgattaccgaagaaatctatcaggttctgtattgcggcaaaaatgcccgtgaagcagcactgaccctgctgggtcgtgcacgtaaagatgaacgtagcagccattaa**ggatcc**

**G3PD*_Cb_*** (restriction enzyme cloning sites in bold; pETCC2)

**catatg**agcaaaatcacctttctgggtggtggtagctttggtagcgcactggcagttctgctggcagaaaaaaacaatgtggtgaacatttatgatcgcgataaaaacgtggtgaacgagatcaacatcaaaaaaaccaacgagaaatatatgaaagattttgccattccgaaaggcgtgaccgcatttaatagcattgaagaggcaattgatggtgccgattatattgttctgagcgttccgagccatgttattcgtagcatgtgtattgccatcaaaggtaaaattccgcgtgatattccgattatcagcattgccaaaggtatcgaagaagataccgataaacgtctgagcgtggttattgaagaagaactggataatccggttgttgtgctgagcggtccgtcacatgccgaagaggttgttatgcgtattccgaccaccattgttagcaccagcaaagaaatgaaatttgcagcagatgttcaggacctgtttatgaccccgtattttcgtgtttataccaacgatgatattatcggtgttgaagttggtggtgccgtgaaaaatgttattgccctggcagccggtgttattgatggtctgggttatggtgataataccaaagcagcactgctgacccgtggtatgaaagaaattagccgtgttggtattgcactgggtggtcgtgcagaaaccttttatggtctgaccggtatgggtgatctgattgttacctgtaccagcatgcatagccgtaatcgtcgtgcaggtctgctgattggtaaaggtatgagcctggaagatgcactgaaagaagttggtatggttgttgaaggtgttaaagcatgtcgtgcattctatcagctgaaagagcgtattggtattagcatgccgattaccgatggtctgtataaaggtctgtttgaaggcaaaaacgccaaaagcattgttgatgaactgatgaaccgtgacaaaaaaagcgaactgttctaa**ggatcc**

**G3PD*_Sc_*** (restriction enzyme cloning sites in bold; pETCC2)

**Catatg**agcgcagcagcagatcgtctgaatctgaccagcggtcatctgaatgcaggtcgtaaacgtagcagcagctcagttagcctgaaagcagcagaaaaaccgtttaaagttaccgttattggtagcggtaattggggcaccaccattgcaaaagttgttgccgaaaattgtaaaggctatccggaagtttttgcaccgattgttcagatgtgggtgtttgaagaagaaatcaacggcgaaaaactgaccgaaattatcaatacccgtcaccagaatgtgaaatatctgcctggtattaccctgccggataatctggttgcaaatccggatctgattgatagcgttaaagatgtggatatcatcgtgtttaacatcccgcatcagtttctgcctcgtatttgtagccagctgaaaggtcatgttgatagccatgttcgtgcaattagctgtctgaaaggttttgaagttggtgcaaaaggtgttcagctgctgagcagttatattaccgaagaactgggtattcagtgtggtgcactgagcggtgcaaatattgcaaccgaagttgcacaagaacattggagcgaaaccaccgttgcatatcacattccgaaagattttcgtggtgaaggtaaagacgttgatcacaaagttctgaaagcactgtttcaccgtccgtattttcatgttagcgttattgaagatgttgccggtattagcatttgtggtgccctgaaaaatgttgttgcactgggttgtggttttgttgaaggtctgggttggggtaataatgcaagcgcagcaattcagcgtgttggtctgggtgaaattattcgttttggccagatgttttttccggaatcacgtgaggaaacctattatcaagaaagtgccggtgttgcagatctgattaccacctgtgccggtggtcgtaatgttaaagttgcacgtctgatggcaaccagcggtaaagatgcatgggaatgcgaaaaagaactgctgaatggtcagagcgcacagggcctgattacctgtaaagaagttcatgaatggctggaaacctgtggtagcgttgaagattttccgctgtttgaagcagtttatcagatcgtgtataacaactatccgatgaaaaatctgccggacatgattgaggaactggatctgcatgaagattaa**ggatcc**

**G3PD*_Af_*** (restriction enzyme cloning sites in bold; pETCC2)

**catatg**attgtgagcattctgggtgccggtgcaatgggtagcgcactgagcgttccgctggttgataatggtaatgaagttcgtatttggggcaccgaatttgataccgaaattctgaaaagcattagcgcaggtcgtgaacatccgcgtctgggtgttaaactgaatggtgttgaaatcttttggcctgagcagctggaaaaatgtctggaaaatgccgaagttgttctgctgggtgtgagcaccgatggtgttctgccggttatgagccgtattctgccgtatctgaaagatcagtatattgtgctgattagcaaaggcctgatcgattttgataatagcgttctgaccgttccggaagcagtttggcgtctgaaacatgatctgcgtgaacgtaccgttgcaattaccggtccggctattgcacgtgaagttgcaaaacgtatgccgaccaccgttgtttttagcagcccgagcgaaagcagcgccaataaaatgaaagaaatcttcgaaaccgagtatttcggtgttgaagttaccaccgatattattggcaccgaaattacctcagccctgaaaaatgtttatagcattgcaattgcctggatccgtggttatgaaagccgtaaaaatgtggaaatgagcaatgccaaaggtgttattgcaacccgtgcaattaatgaaatggccgaactgattgaaatcctgggtggtgatcgtgaaaccgcatttggtctgagcggttttggtgatctgattgcaacctttcgtggtggtcgtaatggtatgctgggtgaactgctgggtaaaggtctgagcattgatgaagcaatggaagaactggaacgtcgtggtgttggtgttgttgaaggttataaaaccgcagaaaaagcatatcgtctgagcagcaaaattaacgcagatacaaaactgctggatagcatttatcgtgtgctgtatgaaggtctgaaagttgaagaagttctgtttgaactggccaccttcaaataa**ggatcc**

**NADH Oxidase Enzymes:**

**Nox*_Ca_*** (restriction enzyme cloning sites in bold; pETCC2)

**catatg**aaaatcgtggtgattggttgtacccatgcaggcaccgcagcagttaaaaccattctgaaagaaaatccggaagccgaaatcaccatttttgaacgcaatgacaacatcagctttctgagctgtggtattgcactgtatgttggtggtgttgttaaagatccggcaggtctgttttatagcaacccggaagaactgagcaaaatgggtgcaaatgtgaaaatcaaacataacgtgaaaagcatcgataccaaaagcaaaaaagtgatcgccgaagatatgaacaccggtgaagaaattgaagtgagctatgataaactggtgaataccaccggtagctggccgattattccgcctattccgggtattgaaagtaaaaacattctgctgtgcaaaaactatgatcaggccaatgttattatccgccagaccaaagatgccaaaaaaatcgttattgtgggtggtggctatattggtattgaactggttgaagcatttcagaaaagcggtaaacaggttaccctgattgatggtctggatcgtattctgaacaaatacctggataaagagttcaccgatatcctggaagatgatctgaaaaaaaacggcattaatctggcactggatcagtgcgtgaaatcctttaaagcaaatgaaaatggcgaagtgaccagcgttgaaaccaccaaaggtgaatatgaagccgatatggttattctgtgtgttggttttcgtccgaataacgaactgctgaaaggtaaagttgatatgctgccgaatgatgccattatcgtggatgaatatatgcgtaccagcgatccggatatttttgcagccggtgatagctgtgcagttcattataatccgaatggcaactatgcctatattccgctggcaaccaatgcagttcgtatgggtatgctgattggcaaaaacattagcaccccgaaagttaaatatcgtggcacccagagcaccagcggtctgaacctgtttggctacaatattggtagtaccggtgttaccgttagtggtgcaccgcagattggtctgaatgttcgtagcgtgattgtgaaagataactaccgtccggaatttatgccgaccaatgaagaaatcattatgcagctggtttatgaagttggcaccaatcgtattgttggcggtcaggttatgagcaaatatgatattacccagagcgcaaataccctgagcctggcaattcagaataaaatgaccattgaggatctggcctatgtggatttcttttttcagccgcattttgatcgtccgtggaactatctgaatattctgggtctggcagcactggaacaagaaggtctgtaa**ggattcc**

**Nox*_Sm_*** (restriction enzyme cloning sites in bold; pETCC2)

**catatg**agcaaaattgttattgtgggtgcaaatcatgcaggcaccgcagcaattaataccattctggataattatggcagcgaaaatgaagtggttgtgtttgatcagaatagcaacattagctttttaggttgtggtatggcactgtggattggcaaacaaattagcggtccgcagggtctgttttatgcagataaagaaagcctggaagcaaaaggtgccaaaatctatatggaaagtccggttaccgccattgattatgatgcaaaacgtgttaccgcactggttaatggtcaagaacatgttgaaagctacgagaaactgattctggcaaccggtagcaccccgattctgcctccgattaaaggtgcagccattaaagaaggtagtcgcgattttgaagcaaccctgaaaaatctgcagttcgtgaaactgtatcagaatgccgaagatgtgattaacaaactgcaggataaaagccagaatctgaatcgtattgcagttgttggtgcaggttatattggtgttgaactggcagaagcatttaaacgtctgggtaaagaagtgattctgattgacgttgttgatacctgtctggcaggttattatgatcaggatctgagcgaaatgatgcgtcagaatctggaagatcatggtatcgaactggcatttggtgaaaccgttaaagcaattgaaggtgatggtaaagtggaacgtattgttaccgataaagcaagccatgatgtggatatggttattctggcagttggttttcgtccgaatacagcactgggtaatgcaaaactgaaaacctttcgtaatggtgcctttctggtggacaaaaaacaagaaaccagcattccggatgtttatgccattggtgattgtgcaaccgtttatgataatgccattaacgataccaactatattgcactggcaagcaatgcactgcgtagcggtattgttgcaggtcataatgcagccggtcataaactggaaagtctgggtgttcagggtagcaatggtatttcaatttttggcctgaatatggttagcaccggtctgacccaagaaaaagccaaacgttttggttataatccggaagttaccgcctttaccgattttcagaaagccagctttatcgagcatgataactatccggttacgctgaaaattgtgtatgataaagatagccgtctggttctgggtgcacagatggccagcaaagaagatatgagcatgggtattcacatgtttagcctggccattcaagagaaagttaccattgaacgtctggccctgctggattatttctttctgccgcattttaatcagccgtacaactatatgaccaaagcagcactgaaagccaagtgataaaagctt**ggatcc**

**Nox*_Ls_*** (restriction enzyme cloning sites in bold; pETCC2)

**catatg**aaagtgattgttgttggttgtacccatgcaggcacctttgcagttaaacagaccattgcagatcatcctgatgcagatgttaccgcctatgaaatgaatgacaacattagctttctgagctgtggtattgcactgtatctgggcaaagaaatcaaaaataacgatccgcgtggcctgttttatagcagtccggaagaactgagcaatctgggtgcaaatgttcagatgcgtcatcaggttaccaatgttgatccggaaaccaaaaccatcaaagtgaaagatctgatcaccaacgaagaaaaaaccgaggcatacgacaaactgattatgaccaccggtagcaaaccgaccgttccgcctattccgggtattgatagcagccgtgtttatctgtgcaaaaactataacgacgccaaaaaactgtttgaagaagcaccgaaagcaaaaacgattaccattattggtagcggttatatcggtgcagaactggcagaagcatatagcaatcagaattacaacgtgaacctgatcgatggtcatgaacgtgttctgtataaatactttgataaagagtttaccgatatcctggccaaagattatgaagcccatggtgttaatctggttctgggtagcaaagttgcagcatttgaagaggttgatgatgaaatcattaccaaaaccctggatggcaaagagattaaaagcgatattgccattctgtgcattggctttcgtccgaataccgaactgctgaaaggtaaagttgcaatgctggataatggtgccattattaccgatgagtatatgcatagcagcaaccgtgatatttttgcagccggtgatagcgcagcagttcattataatccgaccaatagcaatgcatatattccgctggcaaccaatgcagttcgtcagggtcgtctggttggtctgaatctgaccgaagataaagttaaagatatgggcacccagagcagcagcggtctgaaactgtatggtcgtacctatgttagcaccggcattaataccgcactggcaaaagcaaataatctgaaagttagcgaagtgattatcgccgataactatcgtccggaatttatgctgagcaccgatgaagttctgatgagcctggtttatgatccgaaaacccgtgttattctgggtggtgcactgagcagcatgcatgatgttagccagagcgccaatgttctgagcgtttgtattcagaacaaaaacaccattgatgatctggccatggtggatatgctgtttcagccgcagtttgatcgtccgtttaactatctgaatattctgggccaggcagcacaggcacaggcagataaagcacacaaataata**ggatcc**

**Nox*_Ll_*** (restriction enzyme cloning sites in bold; pETCC2)

**catatg**aaaatcgtggtgattggcaccaatcatgcaggtattgcaaccgcaaataccctgctggaacagtatccgggtcatgaaattgttatgattgatcgcaatagcaacatgagctatctgggttgtggcaccgcaatttgggttggtcgtcagattgaaaaaccggatgaactgttttatgccaaagccgaagattttgaagccaaaggtgttaaaattctgaccgaaaccgaagtgagcgaaattgattttgccaacaaaaaagtgtatgccaaaaccaaaagcgacgatgaaattatcgaggcctatgataaactggttctggcaaccggtagccgtccgattattccgaatctgcctggtaaagacctgaaaggtattcactttctgaaactgtttcaagagggtcaggcaattgatgcagaatttgcgaaagaaaaagtgaaacgcattgcagttattggcgcaggttatattggcaccgaaattgccgaagcagcaaaacgtcgtggtaaagaagttctgctgtttgacgcagaaaataccagcctggcaagctattatgatgaagagtttgcaaaaggcatggatgaaaatctggcacagcatggtattgaactgcattttggtgaactggccaaagagtttaaagcaaacgaagagggttatgtgagccagattgttaccaataaagccacctatgatgtggatctggtgattaattgtattggctttaccgccaatagcgcactggcaagcgacaaactggcaacctttaaaaacggtgccatcaaagttgataaacatcagcagagcagcgatccggatgtttatgcagttggtgatgttgcaaccatttatagcaatgcactgcaggattttacctatattgcactggccagcaatgccgttcgtagcggtattgttgcaggtcataatattggtggcaaagaactggaaagcgttggtgttcagggtagcaatggtattagcatttttggctataatatgaccagcaccggtctgagcgttaaagcagccaaaaaactgggtctggaagttagctttagcgattttgaggataaacagaaagcctggtttctgcacgaaaataacgatagcgtgaaaatccgcattgtgtacgaaaccaaatcacgtcgtattattggtgcacagctggcaagcaaaagcgaaattattgcaggcaacatcaacatgtttagcctggccattcaagagaaaaaaaccattgatgaactggcactgctggacctgttttttctgccgcattttaacagcccgtataactatatgaccgttgcagcactgaatgccaaataa**ggatcc**

**Glycerophosphate Oxidase Enzymes:**

**GlpO*Ec*** (restriction enzyme cloning sites in bold; pETCC2)

**catatg**accttcagccagaaagatcgtaaagaaaccattcaagaaaccgccaaaaccacctatgatgtgctgattattggtggtggtattaccggtgccggtgttgcagttcagaccgcagcagcaggtatgaaaaccgttctgctggaaatgcaggattttgcagaaggcaccagcagccgtagcaccaaactggttcatggtggcattcgttatctgaaaacctttgatgttgaagttgttgccgataccgttcgtgaacgtgcaattgttcagcagattgcaccgcatattccgaaaccggatccgatgctgctgccgatttatgatgaaccgggtgcaacctttagcctgtttagcgttaaagttgcaatggatctgtatgatcgtctggcaaatgttaccggtagcaaatatgaaaattatctgctgaccaaagaagaagttctggcacgcgaacctcagctgcaggcagaaaatctggtgggtggtggtgtttatctggattttcgtaataatgatgcccgtctggtgatcgaaaacattaaacgtgcacaggcagatggtgcagcaatgattagcaaagcaaaagttgttggcatcctgcatgatgaacagggcattattaacggtgttgaagtggaagatcagctgaccaatgaacgttttgaagttcatgccaaagtggtgattaatacaaccggtccgtggtcagatattgttcgtcagctggataaaaatgatgaactgcctccgcagatgcgtccgaccaaaggtgttcatctggttgttgatcgtgaaaaactgaaagttccgcagccgacctattttgataccggcaaaaacgatggtcgtatggtttttgttgttccgcgtgagaacaaaacatattttggcaccaccgataccgattataccggtgattttgcacatccgaccgttacccaagaagatgttgattacctgctgacgattgtgaatgaacgctttccgcatgcacagattaccctggatgatattgaagcaagctgggcaggtctgcgtccgctgattaccaataatggtggtagcgattacaatggtggtggcaaaggtaaactgagtgatgaaagctttgagcagattgtggaaagcgtgaaagaatatctggcagatgaacgtcagcgtccggttgttgaaaaagcagttaaacaggcacaagaacgtgttgaagccagcaaagttgatccgagccaggttagccgtggtagcagcctggaacgtagcaaagatggcctgctgaccctggcaggcggtaaaattacagattatcgtctgatggcagaaggtgccgttaaacgtattaatgaactgctgcaagaaagcggtgcatcatttgaactggttgatagcaccacctatccggttagcggtggtgaactggatgcagcaaatgttgaagaagaactggcaaaactggcagatcaggcacagacagcaggttttaatgaagcagcagcaacctatctggcacatctgtatggtagcaatctgccgcaggttctgaactataaaaccaaatttgaaggcctggatgagaaagaaagcaccgcactgaattatagcctgcatgaagaaatggttctgacaccggttgactatctgctgcgtcgtaccaatcatattctgtttatgcgtgatacactggatgatgttaaagcgggtgttgttgcagccatgaccgatttttttggttggagcgaagaagaaaaagccgcacacgttctggaactgaatcaggttattgcagaaagcgatctgacagcactgaaaggtggtaaaaaagatgaataata**ggatcc**

**GlpO*_Mg_***  (Gateway cloning sites in bold; into pDEST17)

**acccagctttcttgtacaaagtggt**tgccgttatcgcaaagcaattaaactgcagaatatgagcatcctgaaaacccattttgatgtggcaattattggcgcaggtattattggtgcaagcattgcttatgaactgagccgttataatctggaagttgtggtgctggaaaaaaatccgaaagttgcaaatgaaaccagcctgggtaatagcggtctgattcatggtggttttgatccggaaccgcataaactggaagcaaaactgaatctgcagggtaatctgaaatggcgtgaatggtttaaacatctggaatttccgcgtgtggaaattgatagcctgattctggcatttaacgaagaagagatgaaacacgttcacatgctgtatgaacgtggtctgaccaatggtctgaacaaaaaagatctgaaagtgctgaccacccaagaagtgctgaaaaaagaaccgaatgttaatccggcagttaaaggtggtctgctgtgtaccagctcagttgcaattcatccggttgaagcaacccgtgcactgctgggtgcagcaaaacagaatgatacccgtctgcgtgttaatagcgaagtgaccaatatcaaatatgaaggtgatcgttttgccctgaccatcaacaacaaatacaaaatctatgcccgtaaagtgattaatgccgcaggtcattatgcagataaactggcaaacaaattcggcttcgatgatttcaaacagaccacccgtcgtggtgaatatcgtattctggataactatgataaaaacctgatcaacagcgtgctgtttaaagttccgaccattcatggtaaaggcattattatcgcaccgaccctggatggtcattatctggttggtccgaccgcacaggatggtgttccgaaagaagatattagcctggtcaccaaagaaaaatatgacctgattggcaaaatcggcaaagatattgttccgagcctgaaaattgaacgcaccattaaaaccattgcaggtagccgtccgattgatgttgaaaccaatgattttgtgatccgcaaaagcaaaaaaaacccgaactttattctggcagcaggtatgcagagtccggcactgagcagcgcaccggcaattgcaagcgaaattgccaatctgctgaatctgaaactgacaccgcgtgaaaatttcaaaccggactataaaatcgacatcttttaatga**acccagctttcttgtacaaagtggt**

**Aldolase Enzyme:**

**FruA*_Sc_*** (restriction enzyme cloning sites in bold; pETCC2)

**catatg**aatcaagagcagttcgacaaaatcaaaaacggcaaaggttttattgcagcactggatcagagcggtggtagcaccccgaaagcactgaaagattatggtgttgaagaaaacgagtatagcaacgacgaagaaatgtttaacctggtgcatgatatgcgtacccgtattattaccagtccggcatttaatggcgaaaaaattctgggtgcaatcctgtttgaacagaccatggatcgtgaagtggaaggtaaatataccggtagctatctggcagataaaggtattgttccgtttctgaaagtggataaaggtctggccgaagaggcagatggtgttcagctgatgaaaccgattccggatctggataaactgctggatcgtgcaaatgaacgtggtatttttggcaccaaaatgcgcagcaatattctggaaaataacaaagaggccatcgaaaaagttgtgaaacagcagtttgaagtggccaaagaaattattgccgcaggtctggttccgattattgaaccggaagttaacattaacgccaaagataaagaagcgattgaagcaaatctggcggaagcaattaaagcggaactggacaatctgaaaaaagaccagtatgtgatgctgaaactgaccattccgaccaaagttaatgcatatagcgaactgattgaacacccgcaggttattcgtgttgttgcactgagtggtggttatagccgtgatgaagcaaacaaaattctgaaacagaacgatggtctgattgcaagctttagccgtgcactggttagcgatctgaatgcacagcagagtgatgcagaatttaacgaaaaactgcaagaagccatcgataccatttttgatgccagcgttaataaagcatga**ggatcc**

1. **Kinetic Data: Supplementary Information**

Kinetic data provided in Tables 2 and 3 was calculated from Michaelis-Menten plots of rate versus substrate concentration for each enzyme, as outlined in the Methods & Materials. The hyperbolic kinetic plots produced from rate data and used to calculate kinetic constants using Hyper^TM^ are provided below, n=3 for each datapoint, with standard deviations given in the calculated constants in the left hand column.

Vmax is given in either mAu/min or µmol/min/mg (as indicated for each graph).

*K*_M_ values are given in mM.

| **Glycerol Kinase** |  |
| --- | --- |
| **GlpK*_Tk_*** | **ATP as substrate** |
| \| Vmax (µmol min^-1^ mg^-1^) = 1875 ± 121.69 \| \| --- \| \| *K*_M_ (mM) = 0.111 ± 0.012 \| | 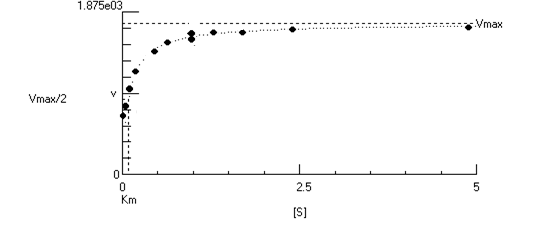 |
|  | **Glycerol as substrate** |
| \| Unweighted \| \| --- \| \| Vmax (µmol min^-1^ mg^-1^) = 1563 ± 57.69 \| \| *K*_M_  (µM) = 15.4 ± 2.36 \| | 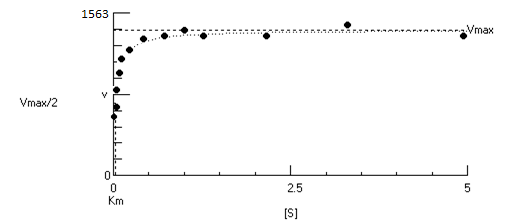 |
| **GlpK*_Cb_*** | **Glycerol as substrate** |
| \| Vmax (µmol min^-1^ mg^-1^) = 25.4 ± 2.149 \| \| --- \| \| *K*_M_ (mM) = 0.356 ± 0.027 \| | 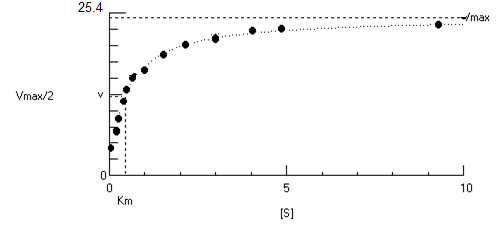 |
|  | **ATP as a substrate** |
| \| Vmax (µmol min^-1^ mg^-1^) = 28.0 ± 3.16 \| \| --- \| \| *K*_M_ (mM) = 0.135 ± 0.016 \| | 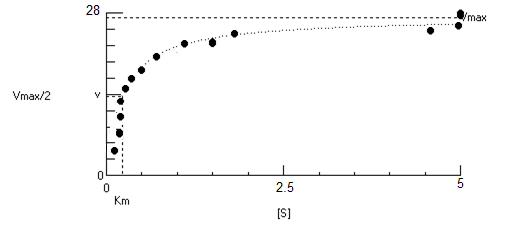 |
| **GlpK*_Bs_*** | **Glycerol as substrate** |
| \| Vmax (µmol min^-1^ mg^-1^) = 38.5 ± 5.6 \| \| --- \| \| *K*_M_ (mM) = 0.153 ± 0.017 \| | 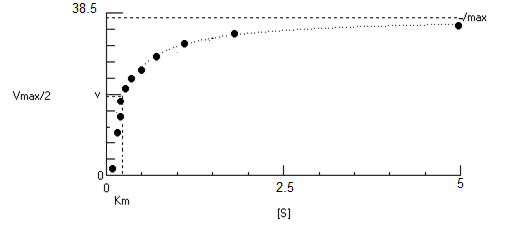 |
|  | **ATP as a substrate** |
| \| Vmax (µmol min^-1^ mg^-1^) = 36.7 ± 5.6 \| \| --- \| \| *K*_M_ (mM) = 0.125 ± 0.011 \| | 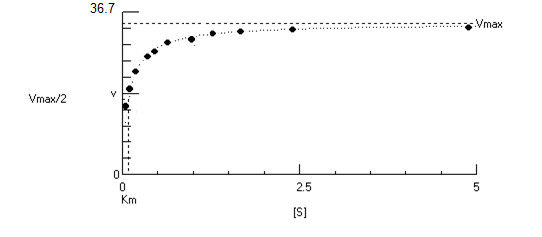 |
|  |  |
| **Acetate Kinase Enzymes** | |
| **AceK*_Ms_*** | **ADP as a substrate** |
| \| Vmax (µmol min^-1^ mg^-1^) = 1129 ± 81.4 \| \| --- \| \| *K*_M_ = 0.113 ± 0.017 \| | 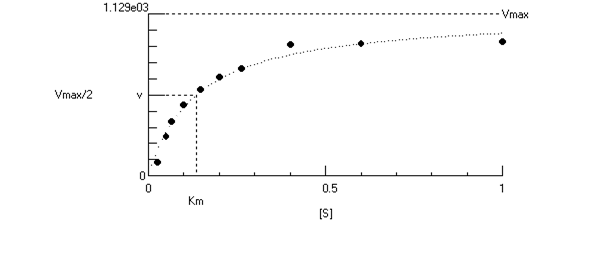 |
|  | **Acetyl phosphate as a substrate** |
| \| Vmax (µmol min^-1^ mg^-1^) = 1123 ± 67.2 \| \| --- \| \| *K*_M_ = 0.390 ± 0.017 \| | 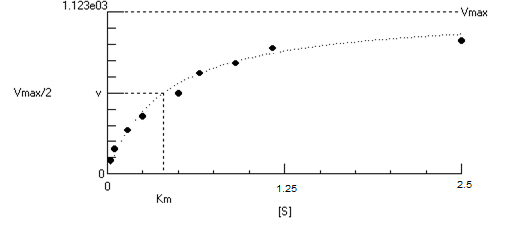 |
| **AceK*_Mt_*** | **ADP as a substrate** |
| \| Vmax (µmol min^-1^ mg^-1^) = 3013 ± 249.2 \| \| --- \| \| *K*_M_ = 0.080 ± 0.007 \| | 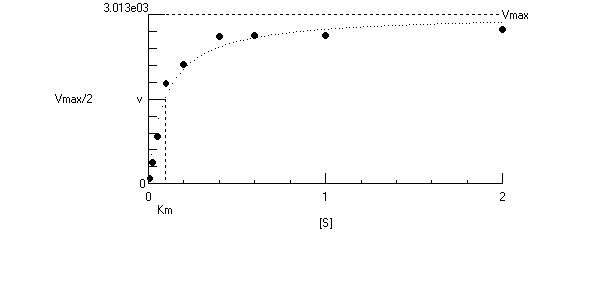 |
|  | **Acetyl phosphate as a substrate** |
| \| Vmax (µmol min^-1^ mg^-1^) = 1193 ± 249.2 \| \| --- \| \| *K*_M_ = 0.470 ± 0.034 \| | 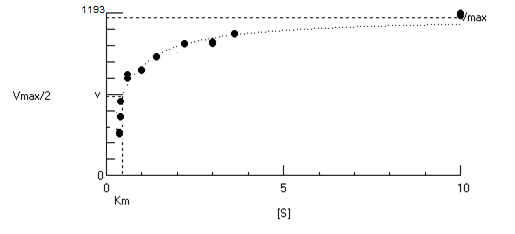 |
| **PyrK*_Bs_*** | **ADP as a substrate** |
| \| Vmax (µmol min^-1^ mg^-1^) = 556..4 ± 3.15 \| \| --- \| \| *K*_M_ = 0.550 ± 0.051 \| | 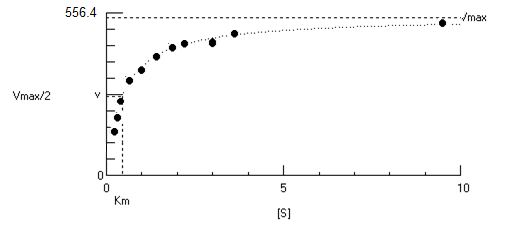 |
|  | **PEP as substrate** |
| \| Vmax (µmol min^-1^ mg^-1^) = 532.1 ± 15.4 \| \| --- \| \| *K*_M_ = 0.110 ± 0.011 \| | 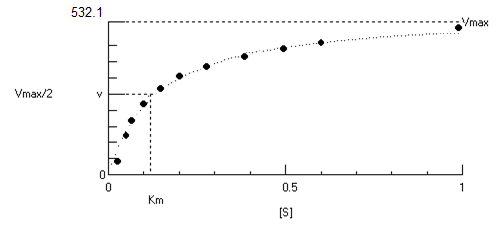 |
|  |  |
| **Glycerol-3-phosphate dehydrogense Enzymes** |  |
| **G3PD*_Ec_*** | **Glycerol-3-phosphate as substrate** |
| \| Vmax (mAu/min)  = 65.3 ± 3.15 \| \| --- \| \| *K*_M_ = 0.059 ± 0.004 \| | 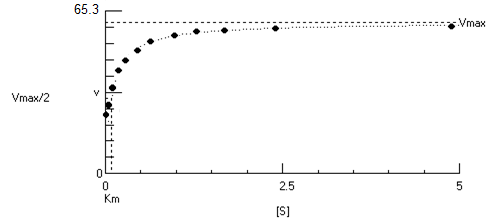 |
|  | **NAD as a substrate** |
| \| Vmax (mAu/min) = 90.99 ± 6.149 \| \| --- \| \| *K*_M_ = 0.158 ± 0.024 \| | 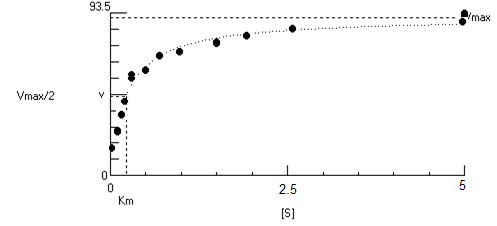 |
| **G3PD*_Ms_*** | **Glycerol-3-phosphate as substrate** |
| \| Vmax (mAu/min)  = 176.2 ± 0.149 \| \| --- \| \| *K*_M_ = 59 ± 5 \| | 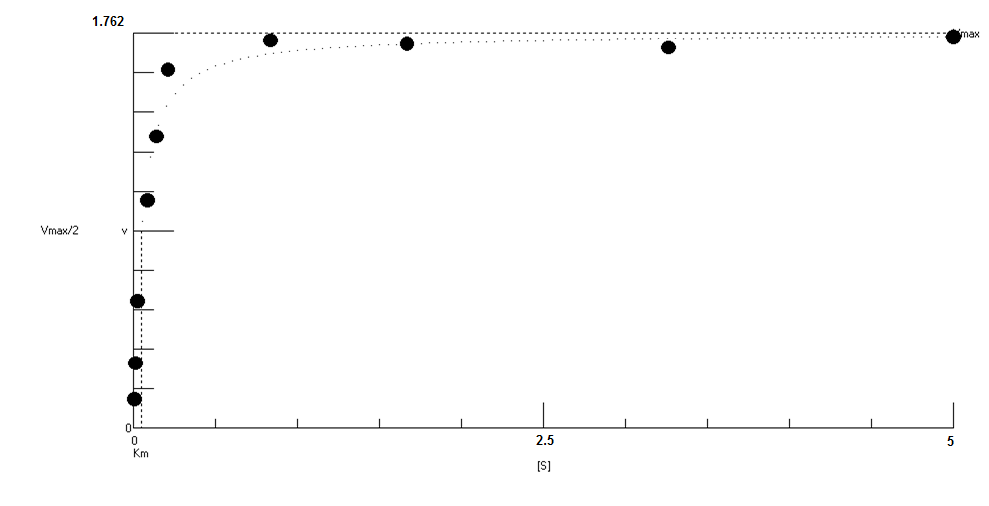 |
|  | **NADP as a substrate** |
| \| Vmax (mAu/min)  = 312 ± 0.149 \| \| --- \| \| *K*_M_ = 258 ± 21 \| | 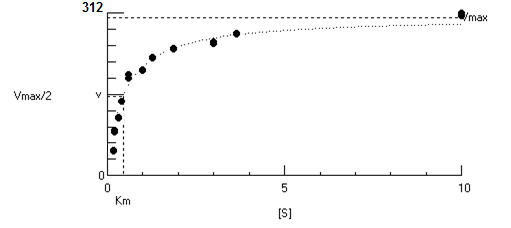 |
| **G3PD*_Af_*** | **Glycerol-3-phosphate as substrate** |
| \| Vmax (mAu/min)  = 53.4 ± 4.15 \| \| --- \| \| *K*_M_ = 111 ± 12 \| | 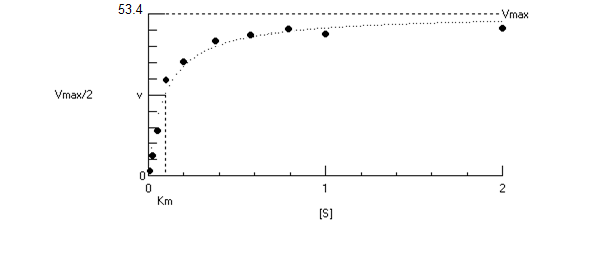 |
|  | **NADP as a substrate** |
| \| Vmax (mAu/min)  = 56.7 ± 5.14 \| \| --- \| \| *K*_M_ = 800 ± 76 \| | 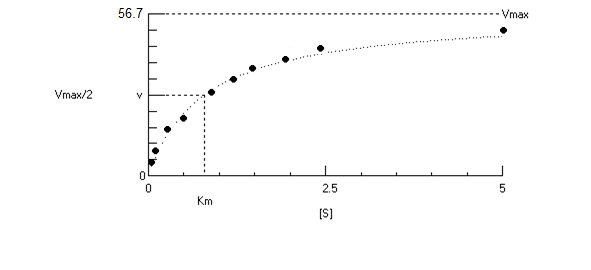 |
|  |  |
| **Glycerophosphate Oxidase Enzymes** | |
| **GlpO*_Mg_*** | **Glycerol-3-phosphate as substrate** |
| \| Unweighted \| \| --- \| \| Vmax (mAu/min)  = 234.3 ± 27.69 \| \| *K*_M_ (µM) = 140.4 ± 13.36 \| \|  \| | **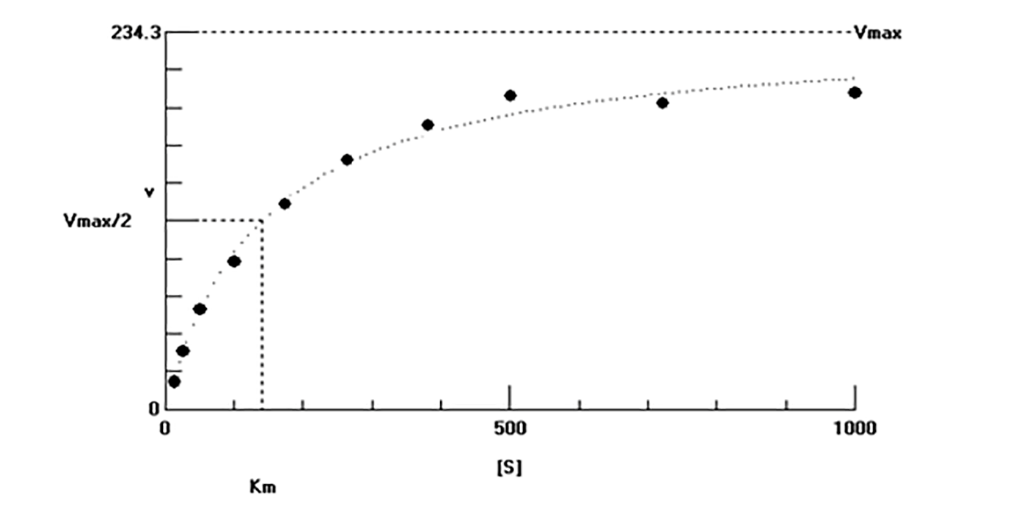** |
| **GlpO*_Ec_*** | **Glycerol-3-phosphate as substrate** |
| \| Unweighted \| \| --- \| \| Vmax (mAu/min)  = 32.1 ± 2.76 \| \| *K*_M_ (µM) = 986 ± 25 \| \|  \| | 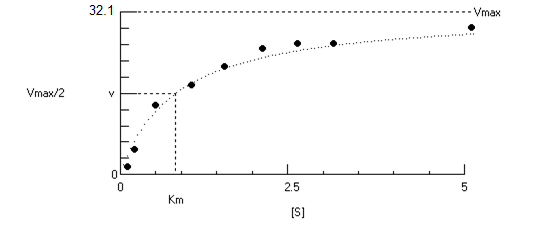 |
| **NADH Oxidase Enzymes** |  |
| **Nox*_Ca_*** | **NADH as substrate** |
| \| Unweighted \| \| --- \| \| Vmax (mAu/min)  = 188.6 ± 17.69 \| \| *K*_M_ (µM) = 258.4 ± 21.36 \| \|  \| | 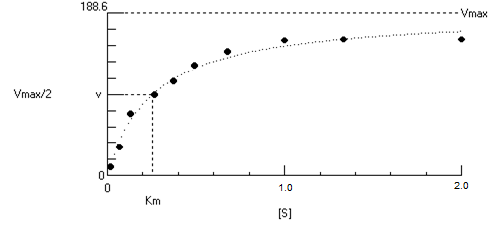 |
| **Nox*_Ls_*** | **NADPH as substrate** |
| \| Unweighted \| \| --- \| \| Vmax (mAu/min)  = 750 ± 17.69 \| \| *K*_M_ (µM) = 6.7 ± 0.9 \| \|  \| | 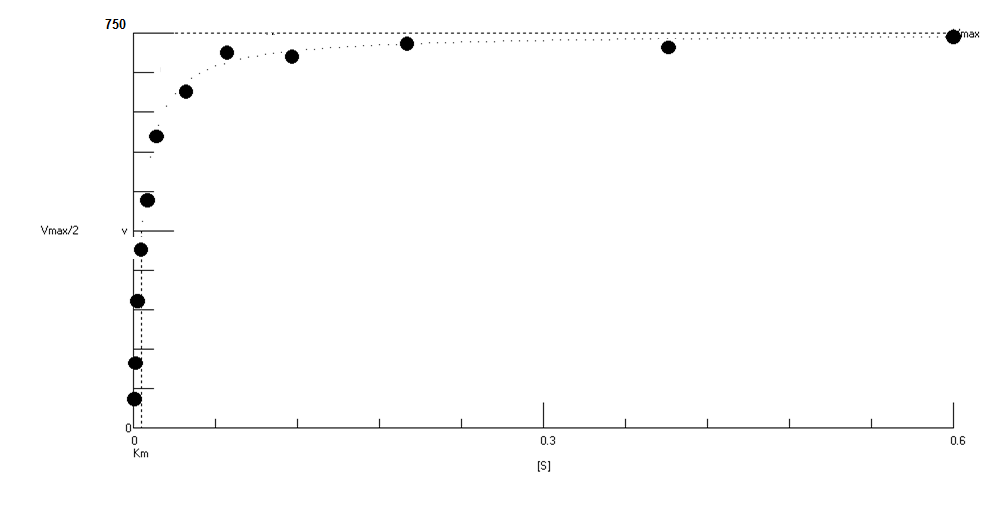 |
|  | **NADH as substrate** |
| \| Unweighted \| \| --- \| \| Vmax (mAu/min)  = 1212 ± 17.69 \| \| *K*_M_ (µM) = 6.1 ± 0.6 \| \|  \| | 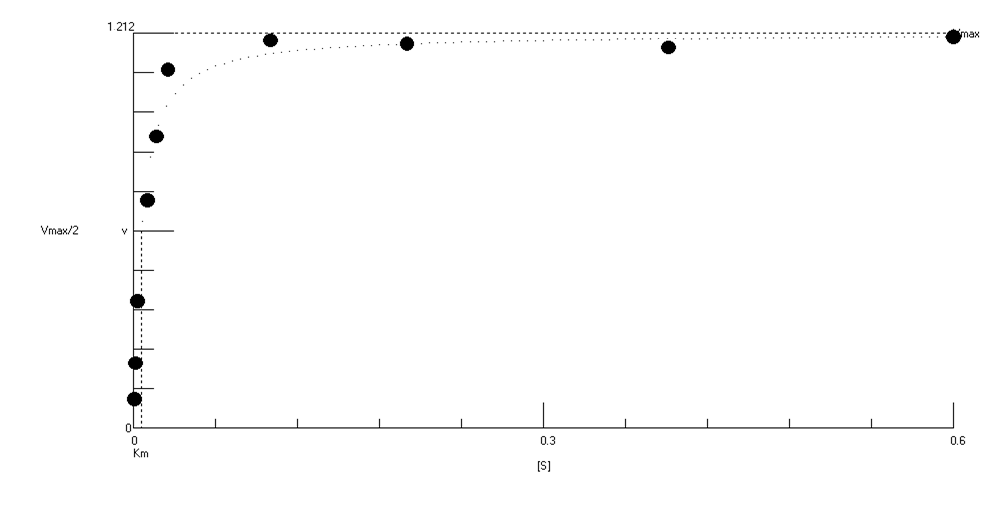 |

1. **Example of the variance in overall production rate.**

**Supplementary Figure S2**. Variance in overall production rate (sum of G3P and DHAP produced from glycerol per second) over time for the optimal four-enzyme cascade comprising GlpK*_Tk_*, AceK*_Ms_*, GlpO*_Mg_* and a commercial catalase enzyme for peroxide mitigation. Reactions were conducted at room temperature in 1 mL total volume with 10 mM glycerol as starting substrate, 100 µM of ATP, 100 µM NAD^+^ or NADP^+^  and 10mM acetyl phosphate co-substrate. Enzymes were added to a final concentration of 14.3, 20.1 and 77.1 pmoles per reaction for GlpK*_Tk_*, AceK*_Ms_*, GlpO*_Mg_* respectively, and to a final concentration of 3U/mL for catalase (Sigma 60634) Samples were collected at various time points and analysed by LCMS. Values summarised in this table are based on 30 minute time point and represent the average of triplicate samples, to the nearest second decimal place. Standard deviation is represented on the chart (only visible for point 1).
